# Crystallization in the Winterbottom Shape and Sharp Fluctuation Laws

**DOI:** 10.1007/s00220-026-05729-8

**Published:** 2026-09-27

**Authors:** Manuel Friedrich, Leonard Kreutz, Ulisse Stefanelli

**Affiliations:** 1 Department of Mathematics, Friedrich-Alexander Universität Erlangen-Nürnberg, Cauerstr. 11, 91058 Erlangen, Germany; 2 Zentrum Mathematik - M7, Technische Universität München, Boltzmannstr. 3, 85748 Garching b. München, Germany; 3 Faculty of Mathematics, University of Vienna, Oskar-Morgenstern-Platz 1, 1090 Vienna, Austria; 4 Vienna Research Platform on Accelerating Photoreaction Discovery, University of Vienna, Wahringerstraße 17, 1090 Wien, Austria

## Abstract

We address finite crystallization in two dimensions in the presence of a flat crystalline substrate. Particles interact through short-range two- and three-body potentials favoring local square-lattice arrangements. An additional interaction term of relative strength β>0 couples the particles and the substrate. Our first main result proves crystallization for all β>0, corresponding to the onset of discrete Winterbottom configurations. The proof relies on a stratification technique from [31], characterizing the topology of the bond graph of minimizing configurations. Our second main result concerns fluctuations estimates for $\beta \in (0,1)$. We obtain bounds on the distance between distinct minimizers with the same number *N* of particles, showing a sharp scaling law $N^{3/4}$ when β is rational, and $N^{1/3}$ when β is irrational and algebraic. This reveals a genuine substrate-driven effect on fluctuation laws. As a corollary, we derive a discrete-to-continuum convergence of minimizers towards the Winterbottom equilibrium shape in the large-particle limit.

## Introduction

At low temperatures, matter typically exhibits crystalline order. In this regime, the interactions between atoms and molecules can be well approximated by configurational potentials. In the zero-temperature limit, it is usually assumed that matter organizes into optimal configurations, minimizing such configurational energies. Proving mathematically that such optimal configurations are periodic (*crystalline*) is precisely the aim of the so-called *crystallization problem*  [34]. Despite considerable attention, rigorous crystallization results remain scarce. To date, crystallization of a finite number of particles (*finite crystallization*) has been established in one and two spatial dimensions, under various assumptions on the interaction potentials, see Sect. 1.1 below.

In this paper, we investigate the *discrete Winterbottom problem*, namely, the determination of equilibrium particle configurations in a crystal in contact with a substrate [60]. Specifically, we establish the first crystallization result in the presence of a flat substrate. Restricting to two space dimensions, we consider a finite system of *N* particles interacting at short range through two- and three-body potentials. These interactions are designed to favor local arrangements with up to four nearest-neighbors bonds forming π/2 angles between them. In addition, the particles interact with a fixed flat crystalline substrate. We are interested in characterizing optimal configurations of such particles. This framework is inspired by epitaxial growth where the crystal develops layer by layer on a substrate. The relative intensity of the particle-particle and particle-substrate interaction is described by a parameter β>0.

Our results are twofold. At first, we prove crystallization for all β>0, see Theorem 2.1. To the best of our knowledge, this constitutes the first two-dimensional finite-crystallization result *relative* to a prescribed substrate. The crystallization proof relies on the *stratification* technique introduced in [31], see Sect. 1.2 below. Due to the substrate interaction, the minimizing *Winterbottom configurations* differ from minimizers in the absence of a substrate, in agreement with the predictions of the Winterbottom problem. Note that for $\beta \ge 1$ the system falls into the so-called *wetting regime*, see Remark 2.3, where minimizers flatten against the substrate and hence are less interesting.

Secondly, for $$\beta \in (0,1)$$ we prove sharp *fluctuations* estimates, see Theorem 2.4. As is typical in crystallization problems, minimizers are nonunique for most values of *N* [18, 48, 56]. We prove matching upper and lower bounds on the distance between minimizers, depending on β. More precisely, if β is rational, we prove that minimizers can differ up to $$ C N^{3/4}$$ particles for some constant C>0. This scaling coincides with that observed in other two-dimensional lattice regimes without a substrate [14, 15, 48, 56]. In contrast, for β irrational but algebraic, minimizers may differ up to $$ C_{\beta , \delta } N^{1/3+ \delta }$$ particles, for arbitrarily small δ>0 with $$C_{\beta ,\delta }\rightarrow \infty $$ as $${ \delta } \rightarrow 0$$. This a genuine effect of the presence of the substrate of fluctuation laws. In a much simplified one-dimensional setting, the sensitivity of ground-state configurations on the value of β has been already observed in [33].

An immediate consequence of our sharp fluctuation results is the characterization of the macroscopic shape of minimizers in the large-system limit $N \rightarrow \infty$. We establish a quantitative discrete-to-continuum convergence of *Winterbottom configurations* towards the corresponding macroscopic *Winterbottom shape*, that is, the equilibrium shape of a macroscopic crystal in contact with the substrate.

### Relation with the literature

In two dimensions, finite crystallization of hard spheres into the triangular lattice was first established by Heitman & Radin [39], building on a result by Harborth [38]. The same result was later reobtained independently by De Luca & Friesecke [17] through an entirely different approach, see Sect. 1.2 below. The case of soft spheres was addressed by Radin [53] and Wagner [59], and subsequently extended by Del Nin & De Luca [16]. Two-body interactions in combination with three-body ones can lead either to the square lattice [48] or the hexagonal one [50], depending on the specific choices. Among these, [31] introduces a new proof technique via stratification, which is the one used in this paper as well, see Sect. 1.2 below. Finite crystallization of ionic dimers systems has been obtained both in the square case [30] and in the hexagonal case [29]. De Luca, Ninno, & Ponsiglione [19] deal with a finite-crystallization setting including orientations. Apart from the very special setting considered in [44], no finite-crystallization result in three dimensions is currently available. To the best of our knowledge, our result in Theorem 2.1 is the first finite-crystallization theorem in which the interaction with a substrate is taken into account.

Note that the crystallization problem takes another flavor in the thermodynamic limit $N \rightarrow \infty$. One-dimensional results can be found in [35, 54, 58], while the stability of periodic one-dimensional configuration, or lack thereof, is discussed in [37]. In two dimensions, Theil [57] proved that certain long-range two-body interactions lead to crystallization in the triangular lattice. Crystallization as $N \rightarrow \infty$ has been obtained by Bétermin, De Luca, & Petrache [8] in the square-lattice case and by Farmer, Esedoḡlu, & Smereka in the hexagonal one [22]. In three dimensions, Flatley & Theil [25] proved that the face-centered-cubic lattice arises when a specific selection mechanism on next-to-nearest neighbors interactions is imposed, see also [24]. A related computation of the corresponding *Wulff shape* for $N\rightarrow \infty$ is in [10]. The defective case has also been considered. The emergence of rigid polycrystaline structures has been tackled in [32]. Dislocations in discrete structures and their coarse-graining have been studied under different assumptions on the interaction energy in [2–4, 36], among others. Yet another different setting is that of lattice-crystallization, where one considers the best lattice for a given lattice energy. Here, the literature is vast and the reader is referred to [6, 7, 12, 13] for references.

Before the present work, the only study dealing with finite crystallization in the presence of a substrate was the already mentioned [33]. There, a one-dimensional hard-sphere finite-crystallization problem is addressed, under the influence of a periodic background modeling substrate interaction. In contrast to the setting considered here, [33] assumes that the substrate and the crystallizing particles favor different lattice parameters.

Uniqueness in finite-crystallization problems occurs only for specific values of *N*. In case of nonuniqueness, the study of the distance of two distinct minimizers (up to lattice translations) has led to different results, depending on the underlying lattice structure. For the triangular lattice, Schmidt [56] proved that minimizers can differ by $CN^{3/4}$ particles, confirming a conjecture from [5]. This has been revisited in [15], where a sharp constant *C* is identified. Cicalese & Leonardi [11] proved the same law by a different approach based on quantitative isoperimetric inequalities [9, 23, 46]. Their method extends to $\mathbb {Z}^d$, as well, providing the upper bound $CN^{1-1/2d}$. This bound, however, is not sharp for d>2, as was shown by identifying the sharp regime, first in the cubic lattice $\mathbb {Z}^3$ [47] and eventually by Mainini & Schmidt [49] in any dimension. The fluctuation law $CN^{3/4}$ has also been shown to hold for the square lattice [48], the hexagonal lattice [14], and ionic dimer systems [29, 30]. To the best of our knowledge, Theorem 2.4 provides the first fluctuation estimates in the presence of a substrate.

Under the assumption of crystallinity, the Winterbottom shape has already been identified in the setting of the discrete double-bubble problem in the square lattice in [26], see also [21], and the continuous counterparts [20, 27, 28]. A discrete-to-continuous justification of the emergence of a hexagonal Winterbottom shape, emerging from a discrete model with two crystals with mismatched lattice parameters, was given by Piovano & Velčić in [51, 52]. The stability of Winterbottom shapes was investigated in [40–42]. In contrast to these works, the novelty of our contribution lies in proving crystallization rather than assuming it. We note, however, that our assumptions on the local geometry of the crystalline lattice are comparatively more stringent.

### Stratification technique

In the two-dimensional case, finite-crystallization for hard spheres has often been obtained by adapting the *induction method over bond-graph layers* by Heitman & Radin [39]. This approach determines the fine geometry of the minimizers by inductively considering the relative effect of boundary vs. bulk particles in the configuration. A second elegant technique to prove finite-cristallizaton for hard spheres has been introduced by De Luca & Friesecke [17], based on *discrete geometry*. Here, a notion of discrete combinatorial curvature is associated to the natural bond graph of the configuration, and a discrete Gauß-Bonnet-like theorem is applied. However, neither of these techniques appears to extend naturally to the case of particle interactions with a substrate. This is particularly evident for the induction method over bond-graph layers [39], relying on the idea that adding (or removing) a boundary layer to a minimizer should maintain minimality – a property which is simply false in the presence of a substrate.

A key technical difference of the present work compared to earlier ones is our use of an alternative argument based on *stratification*. This method, first introduced by the first two authors in [31], provided an alternative proof of the finite-crystallization result for the square lattice of [48].

We now give a heuristic overview of the stratification technique, postponing details to Sect. 3 below. Using purely variational arguments, we first show that minimizers are *regular*: every bond between two neighboring particles has lenght approximately 1, and the angle formed by two adjacent bonds is approximately a multiple of π/2. To each such regular configuration we associate its *strata*, namely all bond paths that are approximately straight. The crystallization result follows by characterizing the topology of these strata. In particular, due to the presence of the substrate, we can distinguish between those strata that are *interacting* or *noninteracting* with it. We prove that two interacting strata, or two noninteracting ones, cannot intersect, and that each interacting stratum crosses all noninteracting ones. This structural property allows us to reconstruct the global topology of the bond graph of minimizers, ultimately proving Theorem 2.1. The stratification approach may be applied to other lattices and higher dimensions, as well. It would also be interesting to consider the case of a nonflat substrate. With respect to our setting, these generalization seem however to imply some relevant technical complications.

### Discrepancy theory

Previous results on fluctuation estimates have relied either on direct manipulations of minimizing configurations [14, 47, 49, 56] or on quantitative discrete isoperimetric inequalities [11, 15, 31, 48] (or both). Extending these techniques to the case of a substrate is not straightforward. On the one hand, the substrate restricts the possibility of directly manipulating minimizers. On the other hand, discrete isoperimetric inequalities in the presence of a substrate are currently not available.

In this paper, we are hence forced to follow a different path, essentially based on *discrepancy theory* for sequences [43]. This plays a crucial role to treat the case $\beta \in (0,1)$ irrational but algebraic. In particular, it is used to prove the following fact: for all $N_0$ large enough, one can find at most $C_{\beta ,\delta } N_0^{2/3+ \delta }$ and at least $c_{\beta ,\delta } N_0^{2/3- \delta }$ minimizers with *N* particles for $N\in [N_0,2N_0]$ taking the form of an exact *rectangle* (i.e., arrangements of particles in $\mathbb {Z}^2$ with equal rows and equal columns). Here, $C_\delta \rightarrow \infty$ and $c_\delta \rightarrow 0$ as $\delta \rightarrow 0$. Moreover, we prove that any two such optimal rectangles differ in the number of particles at least by $c_{\beta ,\delta } N_0^{1/3- \delta }$ and at most by $C_{\beta ,\delta } N_0^{1/3+ \delta }$. As a consequence, all minimizers with $N\in [N_0,2N_0]$ are at most $C_{\beta ,\delta } N_0^{1/3+ \delta }$ far from an optimal rectangle. At the same time, one finds $N\in [N_0,2N_0]$ such that the closest optimal rectangle has distance at least $c_{\beta ,\delta } N_0^{1/3- \delta }$ and uses such a rectangle to the same lower bound on the fluctuation. The case of a nonalgebraic irrational β is not covered by our approach with discrepancy theory, and is hence left open at present.

### Structure of the paper

The model is introduced in Sect. 2, where we also state the main results. In particular, Theorem 2.1 and Theorem 2.4 contain the crystallization and the fluctuation results, respectively. Section 3 presents the stratification technique used in the analysis and applies it to derive both local and global properties of minimizers. The proof of a technical lemma is postponed to the Appendix. Finally, Sect. 4 contains the proof of the main results. Specifically, Theorem 2.1 is proved in Sect. 4.1 and Theorem 2.4 is proved in Sect. 4.2.

## Setting and Main Results

We consider particle systems in two dimensions, and model their interaction by classical potentials in the frame of Molecular Mechanics [1, 45]. Let β>0 be given and define the *substrate* by $\mathcal {L}^-=\mathbb {Z}^2 \cap \{x_2 \le 0\}$. Indicating a *configuration of particles* by $C_N =\{x_1,\ldots ,x_N\} \subset \mathbb {R}^2\cap \{x_2 >0\}$, we define its energy by

**Fig. 1:**
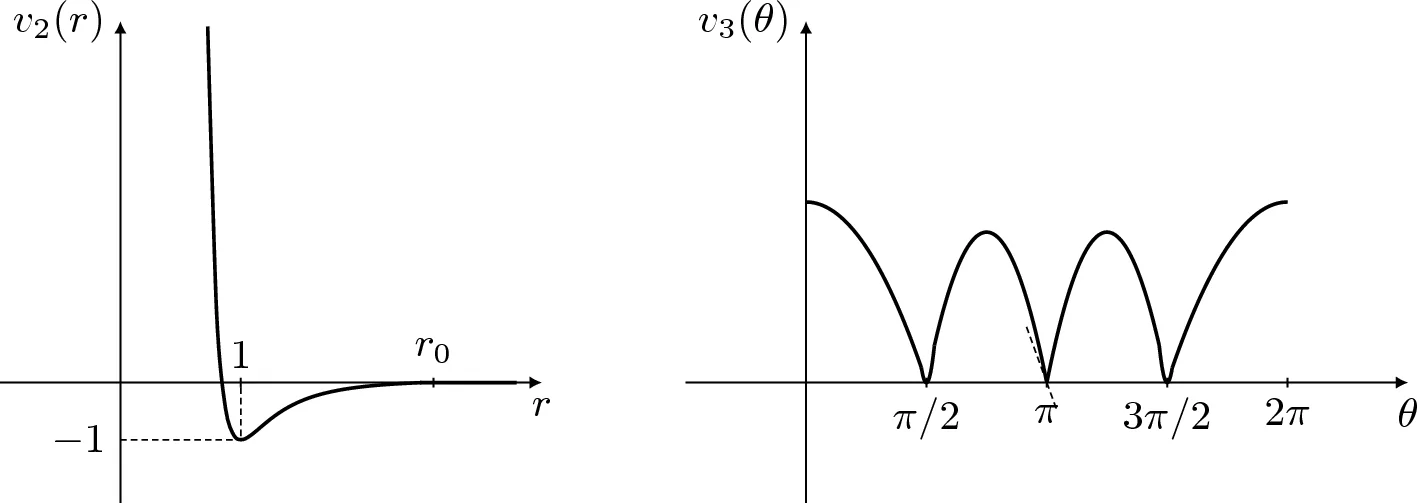
The potentials $v_2$ and $v_3$

2.1 $$\begin{aligned} \mathcal {F}_\beta (C_N) = \frac{1}{2}\underset{x_i \ne x_j}{\sum _{x_i,x_j \in C_N}} v_2(|x_i-x_j|) + \beta \sum _{x_i \in C_N, z \in \mathcal {L}^-} v_2(|x_i-z|) + \frac{1}{2} \sum _{x,y,z } v_3(\theta _{x,y,z})\,, \end{aligned}$$

where $v_2$ and $v_3$ denote two-body and three-body interaction potentials, respectively, which are specified below and depicted in Fig. 1. The third sum runs over triples $(x,y,z)\in ( C_N \cup \mathcal {L}^-)^3$ such that $|x-y|,|y-z|<r_0$ (to be defined later, see ($\mathrm ii_2$)) and $\theta _{x,y,z}$ denotes the angle formed by the vectors x-y and z-y (counted clockwisely). The factor $\frac{1}{2}$ accounts for double counting of bonds and angles. We fix $0<\varepsilon <\varepsilon _0$ for $\varepsilon _0 < \frac{\pi }{6}$ specified in Lemma 3.2 and Remark 3.6. The *two-body potential* $v_2 :[0,+\infty ) \rightarrow {\mathbb {R}} \cup \lbrace + \infty \rbrace$ is asked to satisfy

($$\textrm{i}_2$$): $$\min _{r \ge 0} v_2(r) = v_2(1)=-1$$ and $$v_2(r) >-1$$ if r≠1;
($\textrm{ii}_2$): There exists $1<r_0<\sqrt{2}$ such that $v_2(r)=0$ for all $r \ge r_0$;
($\textrm{iii}_2$): For all $r \in [0,1-\varepsilon ]$ it holds that $v_2(r) > \varepsilon ^{-1}$.

The *three-body potential* $v_3 :[0,2\pi ] \rightarrow \mathbb {R}$ is asked to satisfy

($$\textrm{i}_3$$): $$v_3(\theta )=v_3(2\pi -\theta )$$ for all $$\theta \in [0,2\pi ]$$;
($\textrm{ii}_3$): $v_3(k\pi /2)=0$ for k=1,2,3 and $v_3(\theta ) >0$ if $\theta \notin \{\pi /2,\pi ,3\pi /2\}$;
($\textrm{iii}_3$): $v_3(\theta ) \ge 4(\pi /10 -\varepsilon )^{-1} \, |\theta -\pi |$ for all $\theta \in [\pi - \varepsilon ,\pi + \varepsilon ]$ with equality only if $\theta = \pi$;
($$\textrm{iv}_3$$): If $$\theta \notin [\pi /2-\varepsilon ,\pi /2+\varepsilon ] \cup [\pi -\varepsilon ,\pi +\varepsilon ] \cup [3\pi /2-\varepsilon ,3\pi /2+\varepsilon ]$$, then
  $$\begin{aligned} v_3(\theta ) > \max \{1, 2 \beta \} \frac{4}{(1-\varepsilon )^2}\Big (\sqrt{2} +\frac{1}{2}\Big )^2\,. \end{aligned}$$

We briefly comment on the assumptions. Condition ($\mathrm i_2$) on a unique minimum (here normalized to 1) is natural, e.g., it is valid for Lennard–Jones-type potentials. Assumption ($\mathrm ii_2$) states that $v_2$ has compact support. In particular, it ensures that for configurations $C_N \subset \mathbb {Z}^2$ only particles at distance 1 interact, which are usually referred to as *nearest neighbors* in the literature. Eventually, ($\mathrm iii_2$) prevents clustering of points in the sense that pairs of particles have distance at least 1-ε, see Lemma 3.2 below for details.

Condition ($\mathrm i_3$) ensures that the potential $v_3$ does not depend on how (clockwise or counter-clockwise) bond angles are measured, and ($\mathrm ii_3$) guarantees that for $C_N \subset \mathbb {Z}^2$ there is no contribution stemming from the three-body interaction. Slope conditions similar to ($\mathrm iii_3$) have been used in [29–31, 48, 50] in order to obtain crystallization on the square or hexagonal lattice. As a consequence, the potential is necessarily nonsmooth at π. Let us mention that in the other works (except for [31]) the condition is needed at *all* minimum points of $v_3$, whereas here it is only required at π.

We also point out that in this work the focus lies on devising a general proof strategy for crystallization relative to a substrate, and thus all appearing specific numerical constants are chosen for computational simplicity rather than optimality. The shape of the two potentials $v_2$ and $v_3$ is illustrated in Fig. 1.

Given β>0 and $N \in \mathbb {N}$, it will be convenient to consider the *normalized energy* $2 (\mathcal {F}_\beta (C_N) +2N)$ which essentially counts the number of missing bonds at the free surface of the configuration and the number of bonds between configuration and substrate (plus the angle part, if $C_N \not \subset \mathbb {Z}^2$). In this regard, we further define the *normalized minimal energy*

2.2 $$\begin{aligned} m_\beta (N) = \min _{h \in \{1,\ldots ,N\}} \left( 2h + 2(1-\beta ) \bigg \lceil \frac{N}{h} \bigg \rceil \right) \end{aligned}$$

and the largest *optimal height* by

2.3 $$\begin{aligned} h_*(\beta ,N) =\max \left\{ h \in \{1,\ldots ,N\} :2h + 2(1-\beta ) \bigg \lceil \frac{N}{h} \bigg \rceil =m_\beta (N) \right\} \,. \end{aligned}$$

We now state the main results of the paper.

### Theorem 2.1

(Crystallization). For all β>0 there exists $N_\beta \in \mathbb {N}$ such that for each $C_N \in (\mathbb {R}^2)^N$ with $N \ge N_\beta$ it holds that

2.4 $$\begin{aligned} \mathcal {F}_\beta (C_N) \ge -2N + \frac{1}{2} m_\beta (N)\,. \end{aligned}$$

In case of equality, we have $C_N \subset \mathbb {Z}^2 \cap \{x_2>0\}$ and $\#\left( C_N \cap \{x_2=1\}\right) \ge N/h_*(\beta ,N)$.

**Fig. 2:**
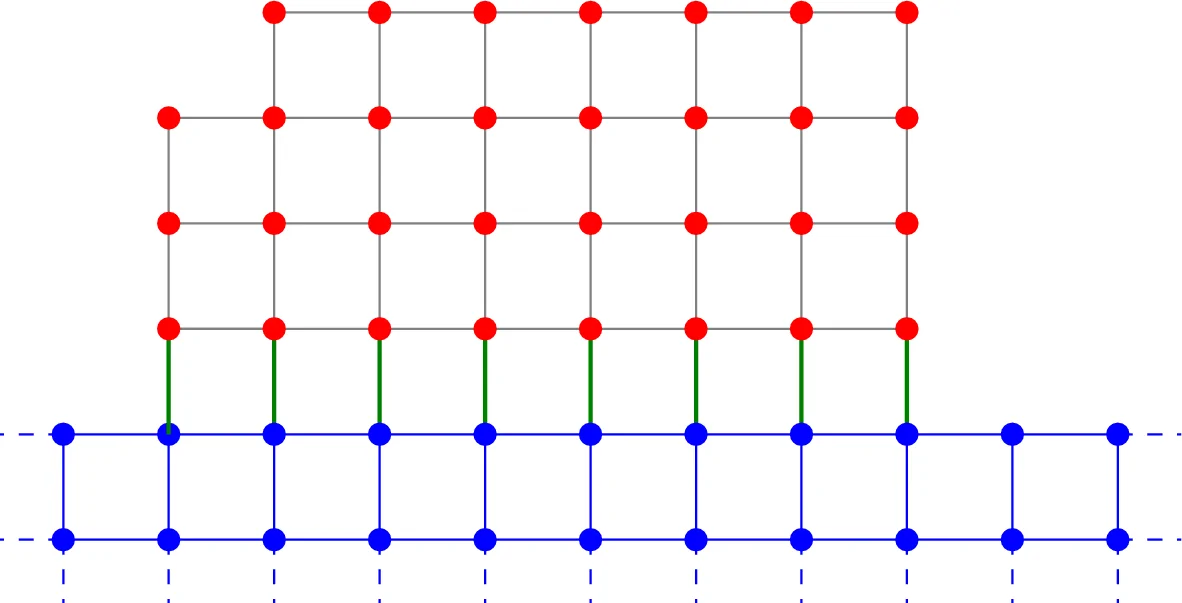
A configuration of minimal energy for N=31 and β=3/7. The bold green bonds indicate the interactions between the substrate and the crystal and the gray bonds indicate the interaction between crystal particles

A configuration of minimal energy is depicted in Fig. 2.

### Remark 2.2

*(Finer characterization of minimizers).* Given $N \ge N_\beta$, minimizers $C_N$ of $\mathcal {F}_\beta$ satisfy some more geometric properties:

(i): $C_N$ is *convex by rows and columns*, i.e., if for some $k \in \mathbb {N}$ and $j \in \{1,2\}$ we have that $x, x+ke_j \in C_N$, then $x+m e_j \in C_N$ for all m=0,…,k.
(ii): Every *column* of $C_N$ is either empty or of the form $C_N \cap (\{k\} \times \mathbb {Z}) = \{1,\ldots ,\alpha _k\}$ for some $\alpha _k \in \mathbb {N}$.
(iii): Setting $l= \#\{k:(\{k\} \times \mathbb {Z}) \cap C_N \ne \emptyset \}$ and $h =\#\{k:(\mathbb {Z}\times \{k\}) \cap C_N \ne \emptyset \}$ we have that
  $$\begin{aligned} \mathcal {F}_\beta (C_N) = - 2N + \frac{1}{2} \big ( 2(1-\beta ) l +2h \big )\,. \end{aligned}$$

### Remark 2.3

*(*$\beta \ge 1$ *: Wetting regime).* If $\beta \ge 1$, it is elementary to check that $h_*(\beta ,N) = 1$. Thus, Theorem 2.1 and Remark 2.2 show that in this case minimizers are horizontal integer shifts of the single chain of particles $\lbrace (i,1) :i = 1,\ldots ,N \rbrace$, and the minimal energy is given by -(N-1)-βN.

In order to state the next theorem, we introduce a *reference rectangle*

$$\begin{aligned} R(\beta ,N) = \left\{ 1,\ldots ,\bigg \lceil \frac{N}{h_*(\beta ,N)}\bigg \rceil \right\} \times \{1,\ldots , h_*(\beta ,N)\} \,. \end{aligned}$$

### Theorem 2.4

(Fluctuations). Let $\beta \in (0,1)$ and $C_N \subset \mathbb {Z}^2 \cap \{x_2>0\}$ be a minimizer of $\mathcal {F}_\beta$. Then the following upper bounds hold true:

($U_1$): If $\beta \in \mathbb {Q}$, then there exists $C_\beta >0$ such that (up to a horizontal translation in $\mathbb {Z} \times \{0\}$)
  $$\begin{aligned} \#\left( C_N \triangle R(\beta ,N) \right) \le C_\beta N^{3/4}\,. \end{aligned}$$
($U_2$): If $\beta \in \mathbb {R}\setminus \mathbb {Q}$ is algebraic, then for every δ>0 there exists $C_{\beta , \delta }>0$ such that (up to a horizontal translation in $\mathbb {Z} \times \{0\}$)
  $$\begin{aligned} \#\left( C_N \triangle R(\beta ,N) \right) \le C_{\beta , \delta } N^{1/3+ \delta }\,. \end{aligned}$$

Moreover, the following lower bounds hold true:

($L_1$): If $\beta \in \mathbb {Q}$, there exists $c_\beta >0$ and a sequence $\{ N_k \}_k \subset \mathbb {N}$ with $N_k \rightarrow \infty$ as $k \rightarrow \infty$ such that for all $k \in \mathbb {N}$ there exists a minimizer of $\mathcal {F}_\beta$, denoted by $C_{N_k}$, satisfying
  $$\begin{aligned} \#\left( C_{N_k} \triangle \left( R(\beta ,N_k) +\tau \right) \right) \ge c_\beta N_k^{3/4}\, \end{aligned}$$
  for all horizontal translations $\tau \in \mathbb {Z} \times \{0\}$.
($L_2$): If $\beta \in \mathbb {R}\setminus \mathbb {Q}$ is algebraic, then for all δ>0 there exists a constant $c_{\beta , \delta }>0$ and a sequence $\{ N_k \}_k \subset \mathbb {N}$ with $N_k \rightarrow \infty$ as $k \rightarrow \infty$ such that for all $k \in \mathbb {N}$ there exists a minimizer of $\mathcal {F}_\beta$, denoted by $C_{N_k}$, satisfying
  $$\begin{aligned} \#\left( C_{N_k} \triangle \left( R(\beta ,N_k) +\tau \right) \right) \ge c_{\beta , \delta } N_k^{1/3- \delta }\, \end{aligned}$$
  for all horizontal translations $\tau \in \mathbb {Z} \times \{0\}$.

**Fig. 3:**
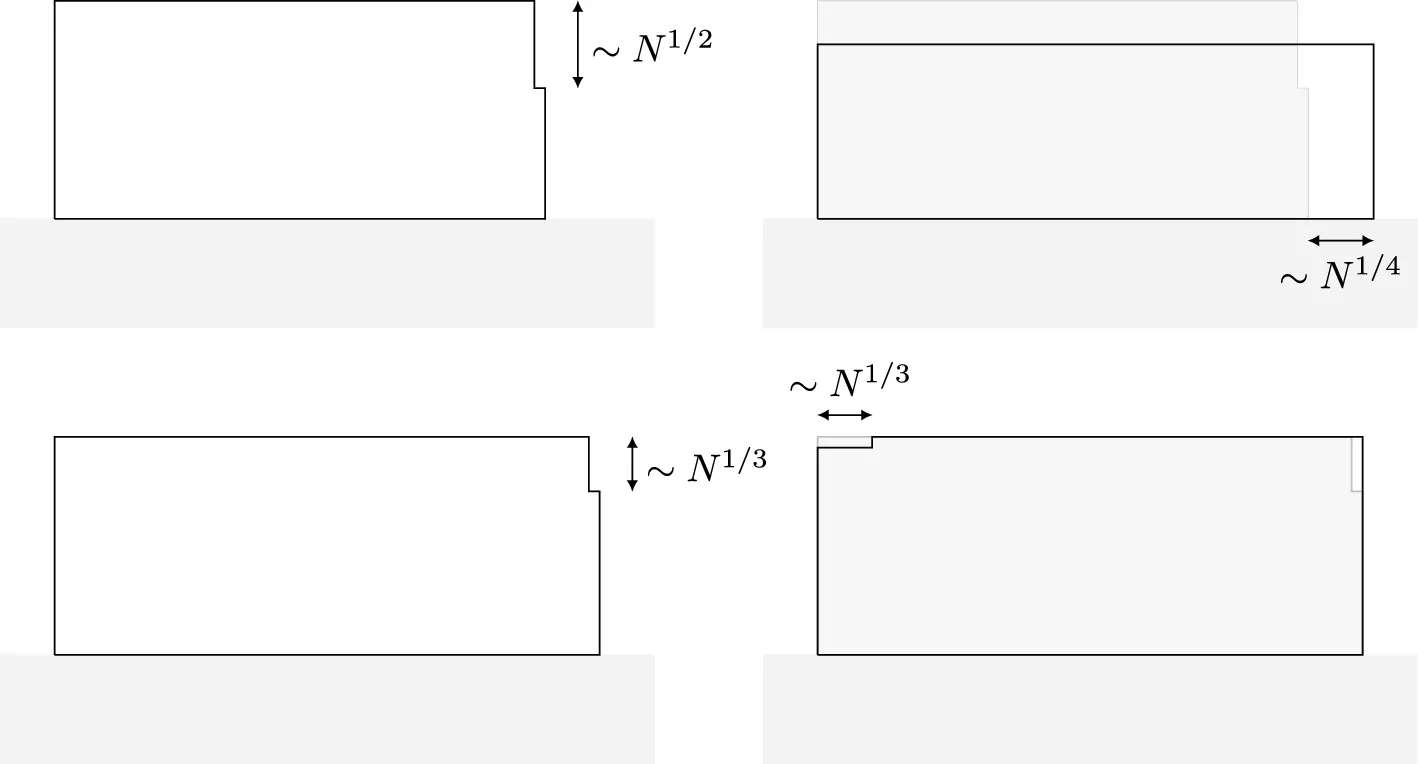
Schematic depiction of ground states that satisfy the optimal fluctuation estimates. The above two for $\beta \in \mathbb {Q}$ and the bottom two for $\beta \in \mathbb {R}{\setminus } \mathbb {Q}$ algebraic

The result provides a fluctuation law of $N^{3/4}$ in the case of rational β and a law of $N^{1/3}$ for irrational, algebraic β. The case of a transcendental interaction parameter is not addressed here. Some configurations satisfying the optimal fluctuation estimates are depicted in Fig. 3.

A direct consequence of the sharp fluctuation estimates of Theorem 2.4 is the characterization of the Winterbottom shape emerging in the large-particle limit $N \rightarrow \infty$. Arguing along the lines of [5] and using also Lemma 4.3 below, one may prove the following.

### Corollary 2.5

(Winterbottom shape). Let $\beta \in (0,1)$. The optimal height $h_*(\beta ,N)$ defined in (2.3) satisfies $N^{-1/2}h_*(\beta ,N) \rightarrow \sqrt{1-\beta }$ as $N \rightarrow \infty$. Letting $C_N =\{x_1^N, \dots , x_N^N\} \subset \mathbb {Z}^2 \cap \{x_2>0\}$ be a minimizer of $\mathcal {F}_\beta$ with $\min \{x_1^j: j=1,\dots ,N\}=1$, and defining the sequence of rescaled empirical measures

$$\begin{aligned} \mu _N:= \frac{1}{N}\sum _{j=1}^N \delta _{x^N_j/\sqrt{N}}\,, \end{aligned}$$

as $N \rightarrow \infty$, the measures $\mu _N$ converge weakly* to the restriction of the Lebesgue in $\mathbb {R}^2$ to the rectangle

$$\begin{aligned} R=\left[ 0,\frac{1}{\sqrt{1-\beta }}\right] \times [0,\sqrt{1-\beta }]\,. \end{aligned}$$

## Stratification

After a short preliminary on graph theory, this section is devoted to detail the main technique of this paper, namely a modification of bond graphs, called *stratification*.

### Bond graph

Let G=(V,E) be a graph, where *V* indicates the set of *vertices* and $E \subset \{\{x,y\} :x,y \in V \text { and } x\ne y \}$ is the set of *edges*. For our purposes, we shall always assume that graphs are embedded in $\mathbb {R}^2$.

For x∈V, we denote the (graph) neighborhood with respect to *G* and the (graph) neighborhood with respect to $\mathcal {L}^-$ by

$$\begin{aligned} \mathcal {N}(x,E) :=\{y \in V :\{x,y\} \in E\}\,, \quad \mathcal {N}_{\mathcal {L}^-}(x) :=\{y \in \mathcal {L}^- :|x-y|\le r_0\}\,, \end{aligned}$$

for $r_0>0$ as given in ($\textrm{ii}_2$). For all such graphs G=(V,E) we define

3.1 $$\begin{aligned} F_\beta (G) = F_{\textrm{bond},\beta }(G) + F_{\textrm{ex},\beta }(G)\,, \end{aligned}$$

where

3.2 $$\begin{aligned} F_{\textrm{bond},\beta }(G) = \sum _{x \in V} \big (4-\#\mathcal {N}(x,E)- 2 \beta \#\mathcal {N}_{\mathcal {L}^-}(x) \big )\, \end{aligned}$$

is the *bond energy* and

$$\begin{aligned} F_{\textrm{ex},\beta }(G) =&\underset{x,y\in V}{\sum _{\{x,y\} \in E}} (v_2(|x-y|)+1) + 2 \beta \sum _{x\in V,y\in \mathcal {N}_{\mathcal {L}^-}(x) } (v_2(|x-y|)+1) \\&+ \sum _{ x,y,z} v_3(\theta _{x,y,z}) \end{aligned}$$

is the *excess energy*. For $V^{\prime }\subset V$, let $G[V^{\prime }]$ be the (vertex) induced subgraph of $V^{\prime }$ in *G*, that is $G\left[V^{\prime }\right]=\left(V^{\prime },E^{\prime }\right)$ with $$E'= \{\{x,y\} \in E:x,y \in V'\}$$. We will identify each configuration $$C_N \subset \mathbb {R}^2$$ with its *natural bond graph* $$G_{\textrm{nat}}=(V,E_{\textrm{nat}})$$, where $$V=C_N$$ and the *natural edges* are given by

3.3 $$\begin{aligned} E_\textrm{nat} = \{\{x,y\} :x,y \in C_N, |x-y|\le r_0\}\,. \end{aligned}$$

This definition is motivated by the relation to the energy $\mathcal {F}_\beta$ defined in (2.1), namely

3.4 $$\begin{aligned} 2\mathcal {F}_\beta (C_N) = -4N + F_\beta (G_{\textrm{nat}})\,. \end{aligned}$$

In Sect. 3.2 below, we will successively modify $E_{\textrm{nat}}$ to a smaller set of edges $E\subset E_\textrm{nat}$, according to a set of given rules.

#### Definition 3.1

We say that G=(V,E) is ε*-regular* for ε>0 small if the following two conditions hold:

(i) For $x,y \in V \cup \mathcal {L}^-$ with x≠y it holds that
  $$\begin{aligned} |x-y| \ge 1-\varepsilon \,; \end{aligned}$$
(ii) For each bond angle $\theta =\theta _{x,y,z}$ with $x,y,z \in V \cup \mathcal {L}^-$ it holds that
  $$\begin{aligned} \theta \in [\pi /2-\varepsilon ,\pi /2+\varepsilon ] \cup [\pi -\varepsilon ,\pi +\varepsilon ] \cup [3\pi /2-\varepsilon ,3\pi /2+\varepsilon ]\,. \end{aligned}$$

Note that, if $G_{\textrm{nat}}=(V,E_{\textrm{nat}})$ is ε-regular, then G=(V,E) is ε-regular for all $E\subset E_{\textrm{nat}}$.

#### Lemma 3.2

There exists $\varepsilon _0 >0$ such that the following holds true: if $v_2$, $v_3$ satisfy ($\textrm{i}_2$)–($\textrm{iii}_2$) and ($\textrm{i}_3$)–($\textrm{iv}_3$) for some $0<\varepsilon <\varepsilon _0$ and if $C_N$ is a minimizer of (2.1), then its natural bond graph $G_{\textrm{nat}}=(V,E_{\textrm{nat}})$ is ε-regular. Moreover, it holds that $\#\mathcal {N}(x,E_{\textrm{nat}}) + \#\mathcal {N}_{\mathcal {L}^-}(x) \le 4$ and $\#\mathcal {N}_{\mathcal {L}^-}(x) \le 1$ for all x∈V.

The proof of this statement is similar to the one of [31, Lemma 3.2]. We include it in Appendix A for convenience of the reader. For the remainder of this paper, we assume that $\varepsilon _0>0$ is chosen small enough such that Lemma 3.2 holds true and that $v_2$ and $v_3$ satisfy $(\textrm{i}_2)$–$(\textrm{iii}_2)$ and $(\textrm{i}_3)$–$(\textrm{iv}_3)$ for some $0<\varepsilon <\varepsilon _0$.

### Stratified bond graph

Given G=(V,E), we say that $\gamma =(x_1,\ldots ,x_n)$ with $x_i \in V$ for all i=1,…,n is a *straight path* if n≥2 and the following holds:

(i) $$\{x_i,x_{i+1}\} \in E$$ for all $$i=1,\ldots ,n-1\,$$;
(ii) $\theta _i \in [\pi -\varepsilon ,\pi +\varepsilon ]$ for all $i \in 2,\ldots ,n-1$, where $\theta _i =\theta _{x_{i+1},x_i,x_{i-1}}$;
(iii) $\{x_i,x_{i+1}\} \ne \{x_{j},x_{j+1}\}$ for all i,j=1,…,n-1, j≠i.

(If n=2, (ii) and (iii) are empty.) The set of straight paths is denoted by

$$\begin{aligned} \Gamma (G) :=\{\gamma \text { straight path}\}\,. \end{aligned}$$

We drop *G* and simply write Γ if no confusion arises. If $\gamma \in \Gamma$ and $x_1=x_n$, we say that γ is *closed* and otherwise that γ is *open*. We define

$$\begin{aligned} {\begin{matrix} & V_i :=\{x \in V :\# \mathcal {N}(x,E) = i \} \text { for } i=0,\ldots ,4 \text {\,,} \\   & V_2^\pi :=\{x \in V_2 :\theta _{x_1,x,x_2}, \in [\pi -\varepsilon ,\pi +\varepsilon ] \text { where } \mathcal {N}(x,E) = \lbrace x_1,x_2 \rbrace \}\,. \end{matrix}} \end{aligned}$$

Note that in the second definition one could equally use the angle $\theta _{x_2,x,x_1}$ as $\theta _{x_2,x,x_1} = 2\pi - \theta _{x_1,x,x_2}$. We define the *set of strata* by

$$\begin{aligned} \mathcal {S}(G):= \mathcal {S}_\Gamma \cup \bigcup _{x \in V_0 \cup V_1 \cup V_2^\pi } s(x), \quad \text {where } \mathcal {S}_\Gamma :=\{\gamma \in \Gamma :\gamma \text { is a maximal element w.r.t.} \subseteq \}\,. \end{aligned}$$

Here, we set $s(x) =\{(x),(x)\}$ for $x \in V_0$ and $s(x) = \{(x)\}$ for $x \in V_1 \cup V_2^\pi$. Adding the *degenerate stratum* (*x*) twice for $V_0$ and once for $V_1 \cup V_2^\pi$ has no geometrical interpretation but is merely convenient to relate the number of strata to $F_{\textrm{bond},\beta }$, see Lemma 3.7 below. In particular, it will ensure that each particle is contained in exactly two strata, see Lemma 3.7(iii).

We say that $s\in \mathcal {S}_\Gamma$, $s= (x_1,\ldots ,x_n)$, is an *interacting stratum* (with the substrate) if there is $z_0 \in \mathcal {N}_{\mathcal {L}^-}(x_1)$ such that $\theta _{z_0,x_1,x_2} \in [\pi -\varepsilon ,\pi +\varepsilon ]$ and/or $z_{n+1} \in \mathcal {N}_{\mathcal {L}^-}(x_n)$ such that $\theta _{x_{n-1},x_n,z_{n+1}} \in [\pi -\varepsilon ,\pi +\varepsilon ]$. The *set of interacting strata* is defined by

$$\begin{aligned} \mathcal {S}_\textrm{int} (G) :=\{ \gamma \in \mathcal {S}_\Gamma :&\gamma \text { interacting stratum}\} \cup \{(x) :x\in V_0 \cup V_2^\pi , \mathcal {N}_{\mathcal {L}^-}(x) \ne \emptyset \}\\  &\cup \{(x) :x \in V_1, \mathcal {N}_{\mathcal {L}^-}(x) \ne \emptyset , x\notin \gamma \text { for each interacting stratum } \gamma \} \,. \end{aligned}$$

**Fig. 4:**
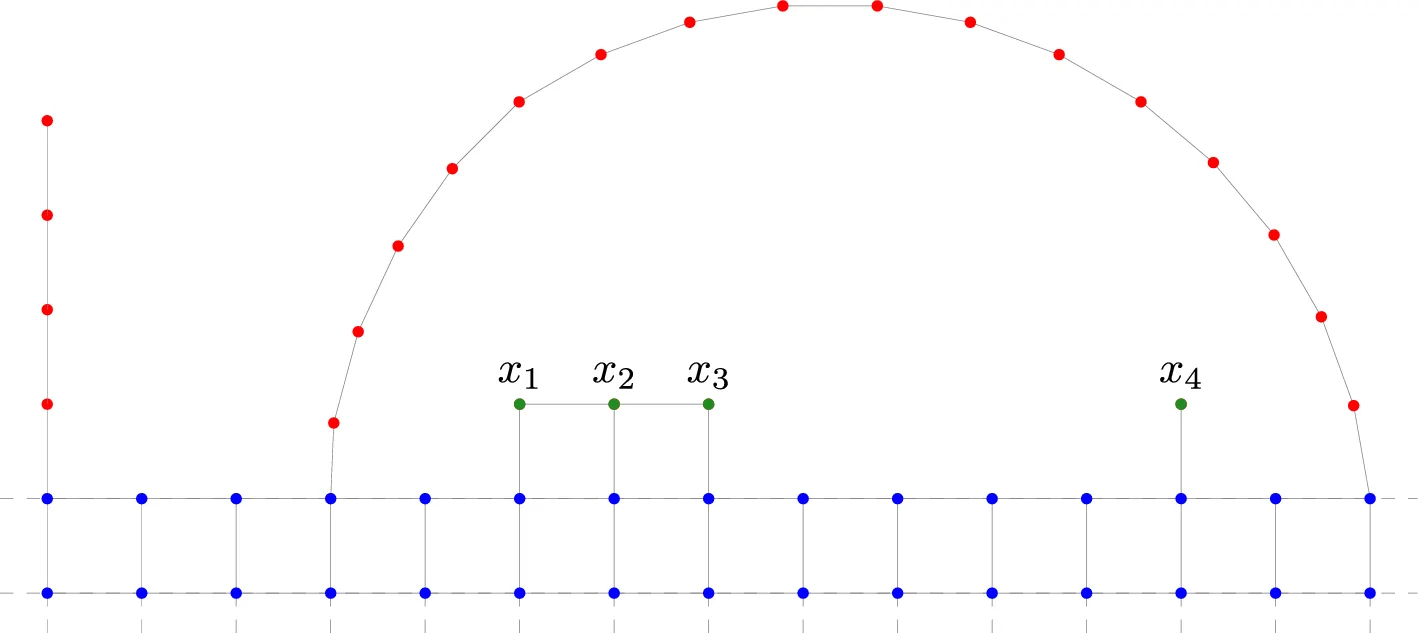
Different interacting strata. Four degenerate ones are indicated in green, where $x_1,x_3 \in V_1$, $x_2 \in V_2^\pi$, and $x_4 \in V_0$

Also here the addition of degenerate strata is convenient to relate the number of strata to $F_{\textrm{bond},\beta }$. The *set of noninteracting strata* is given by $\mathcal {S}_\textrm{no} (G):=\mathcal {S} (G) {\setminus } \mathcal {S}_\textrm{int} (G)$. We say that $s \in \mathcal {S}_\textrm{int} (G)$, $s= (x_1,\ldots ,x_n)$, is *not double touching* if either $\mathcal {N}_{\mathcal {L}^-}(x_1) \ne \emptyset$ or $\mathcal {N}_{\mathcal {L}^-}(x_n)\ne \emptyset$. We drop *G* and write $\mathcal {S}$, $\mathcal {S}_\textrm{int}$, and $\mathcal {S}_\textrm{no}$ if no confusion arises. Different possibilities of interacting strata are illustrated in Fig. 4.

#### Definition 3.3

(Length, orthogonal strata, and span). Let $s \in \mathcal {S}$. By l(s):=#s we denote its *length*. We define the *set of orthogonal strata* to *s* by

$$\begin{aligned} \mathcal {S}^\perp (s)=\{s' \in \mathcal {S} \setminus \{s\} :s\cap s'\ne \emptyset \}\,. \end{aligned}$$

Given $s_0 \in \mathcal {S}$ we define

3.5 $$\begin{aligned} \textrm{span}(s_0) = \bigcup _{s \in \mathcal {S}^\perp (s_0)}s\,. \end{aligned}$$

**Fig. 5:**
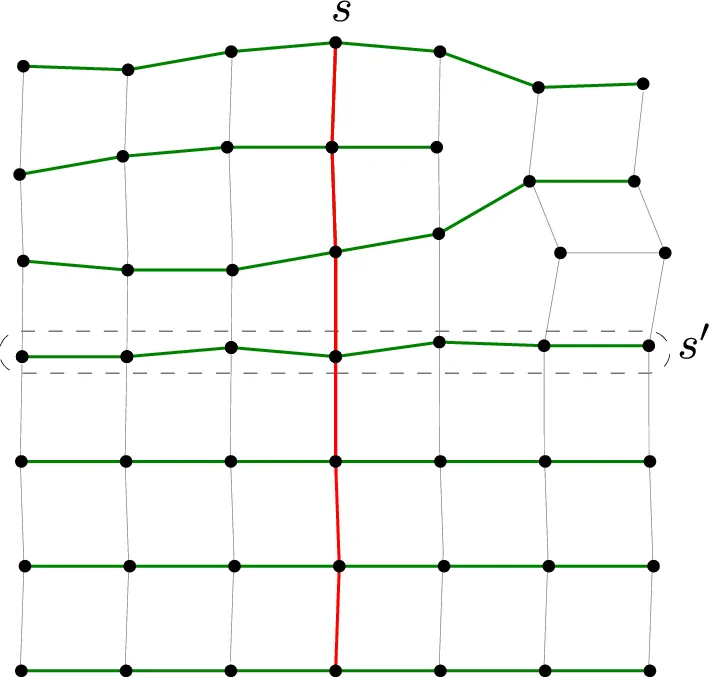
The stratum *s* in red and its orthogonal strata $\mathcal {S}^\perp (s)$ in green. One $s'\in \mathcal {S}^\perp (s)$ is encircled

A stratum $s \in \mathcal {S}$ and its orthogonal strata are illustrated in Fig. 5. We proceed with the definition of the angle excess for straight paths and a corresponding lemma.

#### Definition 3.4

(Angle excess). Given $\gamma =(x_1,\ldots ,x_n) \in \Gamma$, we define the *angle excess* by

$$\begin{aligned} \theta _{{{\text {ex}}}} (\gamma ): = \sum \limits _{{i = 2}}^{{n - 1}} | \theta _{i} - \pi |{\hspace{0.55542pt}} ,\quad {\text {where}}\,\theta _{i} = \theta _{{x_{{i + 1}} ,x_{i} ,x_{{i - 1}} }} . \end{aligned}$$

#### Lemma 3.5

(Small angle excess). Let G=(V,E) be an ε-regular graph. The following implications hold true:

(i): If $\displaystyle \max _{\gamma \in \Gamma } \theta _{\textrm{ex}}(\gamma ) < \frac{3\pi }{2}-\varepsilon$, then all $s \in \Gamma$ are open;
(ii): If $\displaystyle \max _{\gamma \in \Gamma } \theta _{\textrm{ex}}(\gamma ) < \frac{\pi }{2}-\varepsilon$, then $\#\mathcal {S}^\perp (s) = l(s)$ for all $s \in \mathcal {S}$;
(iii): If $\displaystyle \max _{\gamma \in \Gamma } \theta _{\textrm{ex}}(\gamma ) < \frac{\pi }{6}-\varepsilon$, then $s_1 \cap s_2= \emptyset$ for all $s_1,s_2 \in \mathcal {S}^\perp (s)$ and for all $s \in \mathcal {S}$;
(iv): If $\displaystyle \max _{\gamma \in \Gamma } \theta _{\textrm{ex}}(\gamma ) <\frac{\pi }{4}-\frac{5}{2}\varepsilon$ and if $s\in \mathcal {S}_\textrm{int}$, then $s' \in \mathcal {S}_\textrm{no}$ for all $s' \in \mathcal {S}^\perp (s)$;
(v): If $\displaystyle \max _{\gamma \in \Gamma } \theta _{\textrm{ex}}(\gamma ) < \pi -4\varepsilon$, then all $s \in \mathcal {S}_\textrm{int}$ are not double touching;
(vi): If $\displaystyle \max _{\gamma \in \Gamma } \theta _{\textrm{ex}}(\gamma ) <\frac{\pi }{10}-\varepsilon$, if $s_1,s_2 \in \mathcal {S}$ with $s_1 \cap s_2 \ne \emptyset$, and if $s' \in \mathcal {S}^\perp (s_1)$ and $s'' \in \mathcal {S}^\perp (s_2)$, then $\hat{s}_1 \ne \hat{s}_2$ for all $\hat{s}_1,\hat{s}_2 \in \mathcal {S}$ such that $\hat{s}_1 \in \mathcal {S}^\perp (s')$ and $\hat{s}_2 \in \mathcal {S}^\perp (s'')$;
(vii): If $\displaystyle \max _{\gamma \in \Gamma } \theta _{\textrm{ex}}(\gamma ) <\frac{\pi }{10}- \frac{7}{5} \varepsilon$, if $s_1,s_2 \in \mathcal {S}$ with $s_1 \cap s_2 \ne \emptyset$, and if there exists $s' \in \mathcal {S}^\perp (s_1)$ such that $s'\in \mathcal {S}_\textrm{int}$, then $s''\in \mathcal {S}_{\textrm{no}}$ for all $s'' \in \mathcal {S}^\perp (s_2)$.

#### Proof

Statements (i)–(iii) follow as in [31, Proof of Lemma 3.6].

We first prove (iv). Assume that $$s=(x_1,\ldots ,x_n) $$ is interacting, say at $$x_1$$ and $$z_1 \in \mathcal {N}_{\mathcal {L}^-}(x_1)$$, and assume that $$s'=(y_1,\ldots ,y_m)$$ satisfies $$s'\in \mathcal {S}^\perp (s)$$, i.e., $$y_j=x_l$$ for some $$j\in \{1,\ldots , m\}$$ and some $$l \in \{1,\ldots , n \}$$. Assume by contradiction that also $s^{\prime }$ is interacting, say at $$y_m$$ and $$z_k \in \mathcal {N}_{\mathcal {L}^-}(y_m)$$. Consider a straight path $$\gamma _1 = (z_1,\ldots ,z_k)$$ connecting $$z_1$$ and $$z_k$$ in $$\mathcal {L}^-$$, as well as $$\gamma _2 = ( x_1,\ldots , x_l)$$ and $$\gamma _3 = ( y_j,\ldots ,y_m)$$. The closed path (in the sense of graph theory) $$\gamma _1 \circ \gamma _2 \circ \gamma _3$$ forms a (k+l+m-j)-gon. The sum of its interior angles is (k+l+m-j-2)π. In particular, the interior angles at $$z_2,\ldots ,z_{k-1}$$ are π, the interior angles at $$z_1$$ and $$z_k$$ are in $$[\pi /2-\varepsilon ,\pi /2+\varepsilon ]$$, the interior angle at $$x_l$$ is at least $$\pi /2-\varepsilon $$, and the interior angles at $$x_1$$ and $$y_m$$ are in $$[\pi -\varepsilon ,\pi +\varepsilon ]$$. Thus, as $$\theta _{\textrm{ex}}(\gamma _1) =0$$, this implies that $$\theta _{\textrm{ex}}(\gamma _2) + \theta _{\textrm{ex}}(\gamma _3) \ge \frac{\pi }{2} - 5\varepsilon $$. This yields a contradiction since the angle excess of both straight paths is smaller than $$ \frac{\pi }{4} -\frac{5}{2}\varepsilon $$ by assumption.

We now prove (v). Assume by contradiction that $\gamma = (x_1,\ldots ,x_n)$ is double touching, say in $z_1, z_k \in \mathcal {L}^-$. Consider a straight path $(z_1,\ldots ,z_k)$ connecting $z_1$ and $z_k$ in $\mathcal {L}^-$. The path $(x_1,\ldots ,x_n,z_k,z_{k-1}\ldots ,z_1)$ (in the sense of graph theory) forms an (n+k)-gon. The sum of its interior angles is (n+k-2)π. The interior angles at $z_2,\ldots ,z_{k-1}$ are π, the interior angles at $z_1$ and $z_k$ are in $[\pi /2-\varepsilon ,\pi /2+\varepsilon ]$, and the interior angles at $x_1$ and $x_n$ are in $[\pi -\varepsilon ,\pi +\varepsilon ]$. This implies that $\theta _{\textrm{ex}}(\gamma ) \ge \pi -4\varepsilon$, a contradiction.

**Fig. 6:**
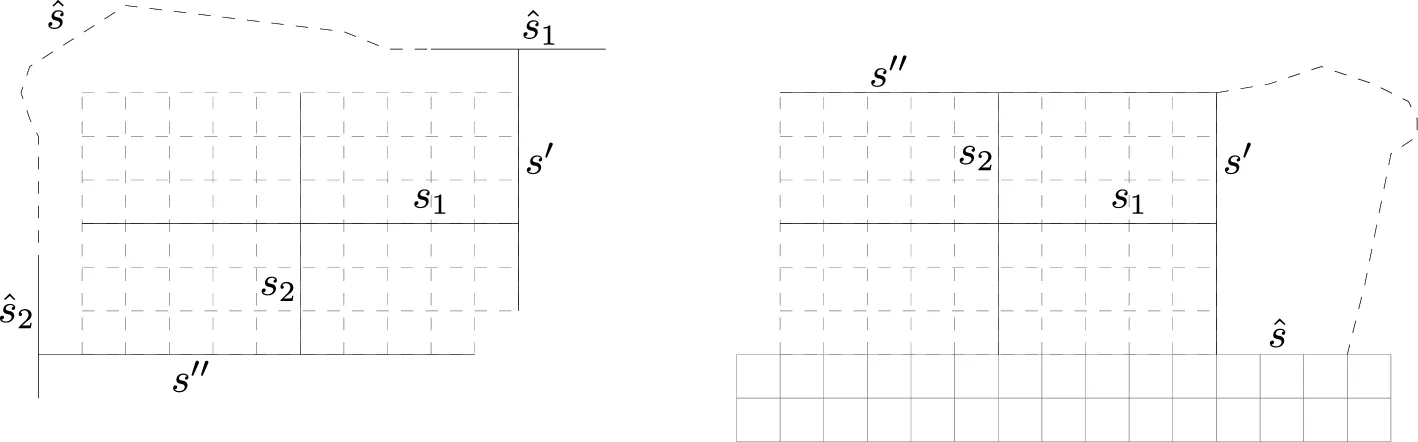
On the left: A configuration that satisfies the contradictory assumption used in the proof of Lemma 3.5(vi). On the right: A configuration that satisfies the contradictory assumption used in the proof of Lemma 3.5(vii)

Next, we prove (vi) and refer to Fig. 6 for an illustration. Assume by contradiction that $\hat{s}_1 = \hat{s}_2 =: \hat{s}$. We denote by $\gamma _1 \subset s_1$ the path connecting the point $s_1 \cap s'$ and the point $s_1 \cap s_2$. Similarly, we denote $\gamma _2 \subset s_2$ the path connecting the point $s_1 \cap s_2$ and the point $s'' \cap s_2$, $\gamma ' \subset s'$ the path connecting the point $s' \cap \hat{s}$ and the point $s' \cap s_1$, $\gamma '' \subset s''$ the path connecting the point $s'' \cap s_2$ and the point $s'' \cap \hat{s}$, and $\hat{\gamma } \subset \hat{s}$ the path connecting the point $s'' \cap \hat{s}$ and the point $s' \cap \hat{s}$. Note that the path $\gamma '\circ \gamma _1 \circ \gamma _2 \circ \gamma '' \circ \hat{\gamma }$ is a closed and thus, taking into account the fact that all bond angles belong to $[k\pi /2-\varepsilon ,k\pi /2+\varepsilon ]$, k=1,2,3, we obtain that $\theta _{\textrm{ex}}(\gamma _1) +\theta _{\textrm{ex}}(\gamma _2) + \theta _{\textrm{ex}}(\gamma ') + \theta _{\textrm{ex}}(\gamma '')+ \theta _{\textrm{ex}}(\hat{\gamma }) \ge \pi /2-5\varepsilon$. This yields a contradiction since the angle excess of all five straight paths is smaller than $\frac{\pi }{10}-\varepsilon$ by assumption.

Lastly, the proof of (vii) follows like the one of (vi) by taking as $$\hat{s} \subset \mathcal {L}^-$$ to be the path connecting $s^{\prime }$ and $s^{\prime \prime }$, see Fig. 6, observing additionally that there are two particles bonded to the substrate where the interior angles of the closed path are in $$[\pi -\varepsilon ,\pi +\varepsilon ]$$, cf. the proof of (v).□

#### Remark 3.6

In the following, we assume that $\varepsilon _0>0$ is small enough such that $\pi /10 - 7\varepsilon /5 \le \pi /6- 4\varepsilon$ for all $0< \varepsilon < \varepsilon _0$.

We state a consequence of Lemma 3.5. (Actually, we only use properties (i), (iv), and (v).)

#### Lemma 3.7

(Graphs with open paths, no double touching). Let G=(V,E) be an ε-regular graph such that $\max _{\gamma \in \Gamma } \theta _{\textrm{ex}}(\gamma ) < \frac{\pi }{10}- \frac{7}{5} \varepsilon$. Then,

(i): $$\sum _{s \in \mathcal {S}} l(s) =2N$$;
(ii): $$ F_\mathrm{bond, \beta } (G) = 2(1-\beta ) \#\mathcal {S}_\textrm{int} + 2\# \mathcal {S}_\textrm{no}$$;
(iii): Each x∈V is contained in exactly two strata *s*(*x*) and $s^\perp (x) \in \mathcal {S}^\perp (s(x))$.

#### Proof

Statement (i) follows exactly as in [31, Proof of Lemma 3.3], so we only need to prove (ii) and (iii).

We first prove (ii). To this end, recalling (3.2) it suffices to prove the claims

$$\begin{aligned} \mathrm{(a)} \quad \#\mathcal {S} = \frac{1}{2}\sum _{x \in V} (4-\#\mathcal {N}(x,E)) \quad \text {and} \quad \mathrm{(b)} \quad \#\mathcal {S}_\textrm{int} = \sum _{x\in V}\#\mathcal {N}_{\mathcal {L}^-}(x)\,. \end{aligned}$$

The proof of (a) can be found in [31, Proof of Lemma 3.3]. We prove (b) by induction over $m :=\#\{x \in V :\mathcal {N}_{\mathcal {L}^-}(x) \ne \emptyset \}$. The statement is clearly true for m=0. Let now m≥1 and fix x∈V such that $\mathcal {N}_{\mathcal {L}^-}(x) \ne \emptyset$. We have that $\#\mathcal {N}_{\mathcal {L}^-}(x)=1$ by Lemma 3.2. Considering $\hat{G}= (V{\setminus } \{x\},E {\setminus } \{\{x,y\} :y \in \mathcal {N}(x,E)\})=(\hat{V},\hat{E})$ we get $\#\{ z \in \hat{V} :\mathcal {N}_{\mathcal {L}^-}(z) \ne \emptyset \} = m-1$. We can apply the induction hypothesis to obtain

$$\begin{aligned} \#\mathcal {S}_\textrm{int}(\hat{G}) = \sum _{z \in \hat{V}} \#\mathcal {N}_{\mathcal {L}^-}(z) = \sum _{z \in V} \#\mathcal {N}_{\mathcal {L}^-}(z) -1\,. \end{aligned}$$

It thus remains to show that $\#\mathcal {S}_\textrm{int}(\hat{G}) = \#\mathcal {S}_\textrm{int}(G)-1$. By definition we clearly have $\mathcal {S}_\textrm{int}(\hat{G}) \subset \mathcal {S}_\textrm{int}({G})$. Consider the unique $s \in \mathcal {S}_\textrm{int}(G)$ with x∈s (uniqueness follows from Lemma 3.5(iv)). It suffices to show that $s {\setminus } \lbrace x \rbrace \notin \mathcal {S}_\textrm{int}(\hat{G})$. If s=(x), this is clear. On the other hand, if $s=(x_1,\ldots ,x_n)$, for n≥2 with $x_1=x$, then, as *s* is not double touching, we have that $(x_2,\ldots ,x_n) \notin \mathcal {S}_\textrm{int}(\hat{G})$. Therefore $\#\mathcal {S}_\textrm{int}(\hat{G}) = \#\mathcal {S}_\textrm{int}(G)-1$.

We now turn to the proof of (iii). Note that, if $x \in V_3\cup V_4 \cup (V_2 {\setminus } V_2^\pi )$, then there exist two straight paths passing through *x* and thus there exist $s_1,s_2 \in \mathcal {S}_\Gamma$ that contain *x*. (Note that all γ are open so the same stratum cannot pass through *x* twice). If $x\in V_2^\pi \cup V_1$, there exists a straight path containing *x* and thus there exists $s \in \mathcal {S}_\Gamma$ containing *x*. By construction, we added the degenerate stratum (*x*) containing *x*. Therefore, also in this case there are two strata $s_1,s_2 \in \mathcal {S}$ containing *x*. Lastly, for $x \in V_0$ we added twice the degenerate stratum (*x*) which contains *x*. This concludes the proof.□

We now come to the *stratification* of bond graphs. The following lemma allows to reduce the problem of crystallization to a purely geometric problem of minimizing the number of strata in graphs containing only open strata with small angle excess. Recall (3.1)–(3.2).

#### Lemma 3.8

(Construction of a graph with small angle excess). Let G=(V,E) be ε-regular. Then, there exists $G_\textrm{o}= (V,E_\textrm{o})$ with $E_\textrm{o} \subset E$ such that

(i): $$\max _{\gamma \in \Gamma (G_\textrm{o})} \theta _{\textrm{ex}}(\gamma ) < \frac{\pi }{10}-7\varepsilon /5$$ ;
(ii): $$G_{\textrm{o}}$$ satisfies
  $$\begin{aligned} F_\beta (G) \ge F_{\textrm{bond},\beta } ( G_{\textrm{o}}) \, \end{aligned}$$
  with equality only if $E = E_\textrm{o}$, |x-y|=1 for all x∈V, $y\in \mathcal {N}(x,E)\cup \mathcal {N}_{\mathcal {L}^-}(x)$, and $\theta \in \{\pi /2,\pi ,3\pi /2\}$ for all $\theta =\theta _{x,y,z}$ with $x,y,z \in V \cup \mathcal {L}^-$ such that |x-y|=|z-y|=1.

#### Proof

The proof can be found in [31, Proof of Lemma 3.7], replacing $\frac{\pi }{6}$ by $\frac{\pi }{10}$. (Compare the assumption in ($\mathrm iii_3$) with the one in [31].)□

We now come to an estimate on $F_{\textrm{bond},\beta }$ for graphs satisfying Lemma 3.8(i). This will be the key ingredient for the proof of Theorem 2.1 in Sect. 4. Recall Definition 3.3.

#### Lemma 3.9

(Estimate on $F_{\textrm{bond},\beta }$, $\beta \in (0,1)$). Let $0<\varepsilon < \varepsilon _0$ and $\beta \in (0,1)$. Let G=(V,E) be an ε-regular graph such that

3.6 $$\begin{aligned} \max _{\gamma \in \Gamma } \theta _{\textrm{ex}}(\gamma ) < \frac{\pi }{10}- \frac{7}{5} \varepsilon \,. \end{aligned}$$

Let $s_1 \in \mathcal {S}$ and $s_2 \in \mathcal {S}^\perp (s_1)$. Then, the following holds true:

(i): $$ F_{\textrm{bond}, \beta } (G) \ge 2(1-\beta ) \max _{s \in s_1,s_2} l(s) + 2\min _{s \in s_1,s_2} l(s)$$ .
(ii): If
  3.7 $$\begin{aligned} \textrm{span}(s_1)\subsetneq V \quad \text {and}\quad \textrm{span}(s_2)\subsetneq V \,, \end{aligned}$$
  then
  $$\begin{aligned} F_{\textrm{bond},\beta } (G) \ge 2(1-\beta ) \max _{s \in s_1,s_2} l(s) + 2\min _{s \in s_1,s_2} l(s) + (4- 2 \beta ) \,. \end{aligned}$$

#### Proof

By (3.6) and Remark 3.6 we observe that all properties in Lemma 3.5 hold. Therefore, also the properties in Lemma 3.7 are satisfied.

*Proof of *(i)*:* First of all, by Lemma 3.5(i) all $s\in \Gamma$ (and thus all $s \in \mathcal {S})$ are open. By Lemma 3.5(iii) we have $\mathcal {S}^\perp (s_1) \cap \mathcal {S}^\perp (s_2)=\emptyset$. Moreover, note that, if there is $s_0 \in \mathcal {S}^\perp (s_1)$ (or $s_0 \in \mathcal {S}^\perp (s_2)$, respectively) such that $s_0 \in \mathcal {S}_\textrm{int}$, then, due to Lemma 3.5(vii), all $\hat{s} \in \mathcal {S}^\perp (s_2)$ (or $\hat{s} \in \mathcal {S}^\perp (s_1)$, respectively) satisfy $\hat{s} \in \mathcal {S}_\textrm{no}$. Together with Lemma 3.7(ii) and Lemma 3.5(ii), this implies

$$\begin{aligned} F_{\textrm{bond},\beta } (G) = 2(1-\beta ) \#\mathcal {S}_\textrm{int} + 2\#\mathcal {S}_\textrm{no} \ge 2(1-\beta )\max _{s \in s_1,s_2} l(s) + 2\min _{s \in s_1,s_2} l(s) \,. \end{aligned}$$

This concludes the proof of (i).

*Proof of *(ii)*:* If (3.7) holds, then we can find $x \in V \setminus \textrm{span}(s_1)$. There are two cases to consider:

- $$x \notin \textrm{span}(s_2)$$;
- $$x \in \textrm{span}(s_2)$$.

*Case *(a)*:* In this case, there are two strata $\hat{s}_1,\hat{s}_2 \in \mathcal {S} {\setminus }\left( \mathcal {S}^\perp (s_1) \cup \mathcal {S}^\perp (s_2)\right)$ such that $x \in \hat{s}_1\cup \hat{s}_2$ and thus $\hat{s}_1 \in \mathcal {S}^\perp (\hat{s}_2)$. By Lemma 3.5(iv) at most one of the two strata $\hat{s}_1, \hat{s}_2$ belongs to $\mathcal {S}_\textrm{int}$. Now, as in the proof of (i), we obtain

$$\begin{aligned} F_{\textrm{bond},\beta } (G) = 2(1-\beta ) \#\mathcal {S}_\textrm{int} + 2\#\mathcal {S}_\textrm{no} \ge 2(1-\beta ) \max _{s \in s_1,s_2} (l(s)+1) + 2\min _{s \in s_1,s_2} (l(s)+1) \,. \end{aligned}$$

This concludes (ii) in case (a).

*Case *(b)*:* As $\textrm{span}(s_2) \subsetneq V$, there exists $z \in V{\setminus } \textrm{span}(s_2)$. We can assume that $z \in \textrm{span}(s_1)$ as otherwise the estimate follows by repeating the argument in (a). Now, due to Lemma 3.5(iii), for $s_1^\perp ,s_2^\perp \in \mathcal {S}^\perp (s_2)$ we have that $s_1^\perp \cap s_2^\perp =\emptyset$, and thus there exists exactly one stratum $s''\in S^\perp (s_2)$ such that $s''\cap \{x\} \ne \emptyset$. In the same fashion, there exists exactly one $s' \in S^\perp (s_1)$ such that $s'\cap \{z\} \ne \emptyset$. We also note that $s\cap \{x\} = \emptyset$ for all $s\in \mathcal {S}^\perp (s_1)$ and $s\cap \{z\} = \emptyset$ for all $s\in \mathcal {S}^\perp (s_2)$ since $x \in V {\setminus } \textrm{span}(s_1)$ and $z \in V{\setminus } \textrm{span}(s_2)$. By Lemma 3.7(iii), for x∈V there exist two strata $\hat{s}_2, s''$ such that $x\in \hat{s}_2, s''$ and therefore (by definition) $\hat{s}_2 \in S^\perp (s'')$. Similarly, there exist two strata $\hat{s}_1, s'$ such that $z \in \hat{s}_1, s''$ and therefore $\hat{s}_1 \in S^\perp (s')$. We get

3.8 $$\begin{aligned} \hat{s}_1, \hat{s}_2 \notin \mathcal {S}^\perp (s_1), \quad \quad \hat{s}_1, \hat{s}_2 \notin \mathcal {S}^\perp (s_2)\,. \end{aligned}$$

Indeed, as $x\in s^{\prime \prime }$ with $$s'' \in \mathcal {S}^\perp (s_2)$$, by Lemma 3.5(iii) we have that $$\hat{s}_2 \notin \mathcal {S}^\perp (s_2)$$ and, by the same reasoning, $$\hat{s}_1 \notin \mathcal {S}^\perp (s_1)$$. As $$x \notin \textrm{span}(s_1)$$, we clearly have that $$\hat{s}_2 \notin \mathcal {S}^\perp (s_1)$$ and analogously $$\hat{s}_1 \notin \mathcal {S}^\perp (s_2)$$.

Moreover, by Lemma 3.5(vi) we have that $\hat{s}_1 \ne \hat{s}_2$, and, if both strata $\hat{s}_1, \hat{s}_2$ belong to $\mathcal {S}_\textrm{int}$, we necessarily have $s', s''\in \mathcal {S}_\textrm{no}$ by Lemma 3.5(iv). Arguing as in the proof of (i) and taking (3.8) into account, we therefore obtain

$$\begin{aligned} F_{\textrm{bond},\beta } (G) = 2(1-\beta ) \#\mathcal {S}_\textrm{int} + 2\#\mathcal {S}_\textrm{no} \ge 2(1-\beta )\max _{s \in s_1,s_2} (l(s)+1) + 2\min _{s \in s_1,s_2} (l(s)+1) \,. \end{aligned}$$

This concludes the proof.□

#### Lemma 3.10

(Estimate on $F_{\textrm{bond},\beta }$, $\beta \ge 1$). Let $0<\varepsilon < \varepsilon _0$ and $\beta \ge 1$. Let G=(V,E) be an ε-regular graph satisfying (3.6). Then, $F_{\textrm{bond}, \beta } (G) \ge 2(1-\beta ) N + 2$ with equality if and only if $\#\mathcal {S}_\textrm{int} = N$.

#### Proof

Lemma 3.7(iii) and Lemma 3.5(iv) clearly imply $\#\mathcal {S}_\textrm{int} \le N$ and $\#\mathcal {S}_\textrm{no} \ge 1$. This along with the identity $F_{\textrm{bond},\beta } (G) = 2(1-\beta ) \#\mathcal {S}_\textrm{int} + 2\#\mathcal {S}_\textrm{no}$ from Lemma 3.7(ii) yields the statement.□

## Proof of the Main Results

This section is devoted to the proofs of Theorem Theorem 2.1 and Theorem 2.4.

### Crystallization

For the proof of Theorem Theorem 2.1, our strategy is to show that the minimum of $F_\beta$ is given by $m_\beta (N)$, and that it is attained by subsets of $\mathbb {Z}^2$ that touch the substrate. In view of (3.4), this will show the result. Recall the definition of $G_{\textrm{nat}}$ in (3.3). We first state the following upper bound.

#### Lemma 4.1

(Upper bound). Let $C_N$ be a minimizer of (2.1). Then, $G_{\textrm{nat}}$ satisfies

$$\begin{aligned} F_\beta (G_{\textrm{nat}}) \le m_\beta (N)\,. \end{aligned}$$

#### Proof

Fix $N \in \mathbb {N}$ and let $h \in \{1,\ldots ,N\}$ be such that $2h+ 2(1-\beta ) \big \lceil \frac{N}{h}\big \rceil = m_\beta (N)$. We have two cases:

(i) $\frac{N}{h} \notin \mathbb {N}$. In this case, $N=h\lfloor \frac{N}{h}\rfloor +k$ for some $k \in \{1,\ldots ,h-1\}$, and we define
  $$\begin{aligned} \bar{C}_N =\left( \left\{ 1,\ldots ,\bigg \lceil \frac{N}{h}\bigg \rceil -1\right\} \times \{1,\ldots ,h\}\right) \cup \left( \left\{ \bigg \lceil \frac{N}{h}\bigg \rceil \right\} \times \{1,\ldots ,k\}\right) \,. \end{aligned}$$
(ii) $\frac{N}{h} \in \mathbb {N}$. Here, one chooses $\bar{C}_N = \{1,\ldots \frac{N}{h}\} \times \{1,\ldots ,h\}$.

In view of (3.2), by directly checking that $F_\beta (\bar{G}_\textrm{nat} ) =F_{\textrm{bond},\beta }(\bar{G}_\textrm{nat}) = 2\,h+ 2(1-\beta ) \big \lceil \frac{N}{h}\big \rceil = m_\beta (N)$, where $\bar{G}_\textrm{nat}$ denotes the natural bond graph related to $\bar{C}_N$, one concludes the proof.□

Given the upper bound of Lemma 4.1, the proof of Theorem 2.1 follows by checking the corresponding lower bound.

#### Proof of Theorem 2.1

Let $C_N$ be a minimizer of (2.1). Then, its natural bond graph $G_{\textrm{nat}}$ is ε-regular by Lemma 3.2. We denote by $G_\textrm{o}=(V,E_\textrm{o})$ the graph obtained in Lemma 3.8. The graph $G_\textrm{o}$ is also ε-regular and satisfies

$$\begin{aligned} \max _{\gamma \in \Gamma (G_o)}\theta _{\textrm{ex}}(\gamma ) < \pi /10- 7\varepsilon /5\,, \end{aligned}$$

i.e., all properties derived in Sect. 3.2 hold for the graph $G_\textrm{o}$. The main part of the proof consists in verifying

4.1 $$\begin{aligned} F_{\textrm{bond},\beta }( G_{\textrm{o}} ) \ge m_\beta (N)\,. \end{aligned}$$

Once (4.1) is proven, we conclude as follows. First, (2.4) holds due to Lemma 3.8(ii) and (3.4). To characterize the equality case, we get from Lemma 3.8 that $G=G_\textrm{o}$, that all bond lengths are 1 (including the ones between *V* and $\mathcal {L}^-$), and all bond angles belong to $\{\pi /2,\pi ,3/2\pi \}$. This shows that each connected component (in the sense of graph theory) of *G* lies in a rotated and shifted version of $\mathbb {Z}^2$. If there was more than one connected component, one could obtain a modified configuration with an additional bond. This contradicts minimality. Furthermore, if $\mathcal {S}_\textrm{int} =\emptyset$, one could obtain a modified configuration with strictly less energy than the original one by placing a rotated and shifted version of *G* in such a way that $V \subset \mathbb {Z}^2$ and $\mathcal {N}_{\mathcal {L}^-}(x) \ne \emptyset$ for some x∈V. As a consequence, we get that $V\subset \mathbb {Z}^2 \cap \lbrace x_2>0 \rbrace$ with length $l= \#\{k:(\{k\} \times \mathbb {Z}) \cap V \ne \emptyset \}$ and height $h =\#\{k:(\mathbb {Z}\times \{k\}) \cap V \ne \emptyset \}$, where the properties stated in Remark 2.2(i),(ii) follow by repeating the argument in [48, Proposition 6.3]. This also shows Remark 2.2(iii) and eventually the remaining property $\#(V \cap \{x_2=1\}) \ge N/ h_*(\beta ,N)$.

We now show (4.1). In the following, for simplicity we write $\mathcal {S}$, $\mathcal {S}_\textrm{int}$, and $\mathcal {S}_\textrm{no}$ in place of $\mathcal {S}(G_{\textrm{o}})$, $\mathcal {S}_\textrm{int}(G_{\textrm{o}})$, and $\mathcal {S}_\textrm{no}(G_{\textrm{o}})$. The case $\beta \ge 1$ directly follows from Lemma 3.10, so we focus on the case $\beta \in (0,1)$. Recalling (3.5) we distinguish two cases:

(i) There exists $s\in \mathcal {S}$ such that
  4.2 $$\begin{aligned} \textrm{span}(s) =V\,; \end{aligned}$$
(ii) For all $s \in \mathcal {S}$ we have
  4.3 $$\begin{aligned} \textrm{span}(s) \subsetneq V\,. \end{aligned}$$

*Case (i):* Select $s\in \mathcal {S}$ such that (4.2) holds true. By (4.2) and Lemma 3.5(ii),(iii), choosing some $\hat{s} \in \textrm{argmax}_{s' \in \mathcal {S}^\perp (s)}\, l(s')$ we get $l(s)\cdot l(\hat{s}) \ge \sum _{s' \in \mathcal {S}^\perp (s)}\, l(s') = N$. Assume without loss of generality that $l(\hat{s}) \ge l(s)$. Then, $l(\hat{s}) \ge \lceil N/l(s)\rceil$. Using Lemma 3.9(i), we obtain

$$\begin{aligned} F_{\textrm{bond},\beta }( G_{\textrm{o}}) \ge 2l(s) + 2(1-\beta ) l(\hat{s}) \ge 2 l(s) + 2(1-\beta ) \lceil N/l(s)\rceil \ge m_\beta (N)\,. \end{aligned}$$

This concludes Case (i).

*Case (ii):* Our goal is to find $N_\beta$ such that for all $N \ge N_\beta$ the estimate

4.4 $$\begin{aligned} 2\#\mathcal {S}_\textrm{no} + 2(1-\beta )\#\mathcal {S}_\textrm{int} = F_{\textrm{bond},\beta }( G_{\textrm{o}} ) > m_\beta (N) \end{aligned}$$

holds which shows that this case can never occur for a minimizer. Define $p :\mathcal {S} \rightarrow \lbrace \frac{1}{2}, \frac{1}{2(1-\beta )} \rbrace$ by

4.5 $$\begin{aligned} p(s) = {\left\{ \begin{array}{ll} \frac{1}{2} & \text {if } s \in \mathcal {S}_\textrm{no}\,,\\ \frac{1}{2(1-\beta )} & \text {if } s \in \mathcal {S}_\textrm{int}\,.\\ \end{array}\right. } \end{aligned}$$

We prove that there exists $x_0 \in V$ such that

4.6 $$\begin{aligned} p(s(x_0)) l(s(x_0)) + p(s^\perp (x_0)) l(s^\perp (x_0)) \ge F_{\textrm{bond},\beta }(G_{\textrm{o}})^{-1} 4N\,, \end{aligned}$$

where $s(x_0),s^\perp (x_0)\in \mathcal {S}$ are the two strata such that $x_0 \in s(x_0)\cap s^\perp (x_0)$, see Lemma 3.7(iii). To this end, define $\mu (s) = \frac{1}{p(s)}$. By Lemma 3.7(ii) we have

4.7 $$\begin{aligned} \mu (\mathcal {S}):= \sum _{s \in \mathcal {S}} \mu (s) = 2\#\mathcal {S}_\textrm{no} + 2(1-\beta ) \#\mathcal {S}_\textrm{int}=F_{\textrm{bond},\beta }(G_{\textrm{o}})\,. \end{aligned}$$

Now, by Jensen’s inequality together with Lemma 3.7(i) and (4.7) we obtain

4.8 $$\begin{aligned} \sum _{s\in \mathcal {S}} \left( p(s)l(s)\right) ^2\mu (s)&\ge \mu (\mathcal {S})^{-1} \left( \sum _{s\in \mathcal {S}} p(s)l(s)\mu (s)\right) ^2 = \mu (\mathcal {S})^{-1} \left( \sum _{s\in \mathcal {S}}l(s)\right) ^2 \nonumber \\  &= \mu (\mathcal {S})^{-1} 4N^2 = F_{\textrm{bond},\beta }(G_{\textrm{o}})^{-1} 4 N^2\,. \end{aligned}$$

On the other hand, letting $s(x),s^\perp (x)\in \mathcal {S}$ be the two strata such that $x \in s(x)\cap s^\perp (x)$, exchanging the order of summation, and using Lemma 3.7(iii), we obtain

4.9 $$\begin{aligned} {\begin{matrix} \sum _{s\in \mathcal {S}} \left( p(s)l(s)\right) ^2\mu (s) & = \sum _{s\in \mathcal {S}} p(s)l(s)^2 = \sum _{s \in \mathcal {S}} \sum _{x \in s} p(s)l(s) = \sum _{s \in \mathcal {S}} \sum _{x \in V} p(s)l(s) \textrm{1}_s(x) \\   & = \sum _{x \in V} \sum _{s \in \mathcal {S}} p(s)l(s) \textrm{1}_s(x) = \sum _{x\in V} \big (p(s(x))l(s(x)) + p(s^\perp (x))l(s^\perp (x)) \big )\,. \end{matrix}} \end{aligned}$$

Inequality (4.6) follows from (4.8) and (4.9) by selecting $x_0 \in V$ such that $p(s(x_0))l(s(x_0)) + p(s^\perp (x_0))l(s^\perp (x_0)) \ge p(s(x))l(s(x)) + p(s^\perp (x))l(s^\perp (x))$ for all x∈V. Next, using (4.3) and Lemma 3.9(ii) for $s(x_0)$ and $s^\perp (x_0)$, we obtain

$$\begin{aligned} F_{\textrm{bond},\beta }(G_{\textrm{o}}) \ge 2(1-\beta ) \max _{s \in s(x_0),s^\perp (x_0)} l(s) + 2 \min _{s \in s(x_0),s^\perp (x_0)} l(s) + (4- 2 \beta )\,. \end{aligned}$$

Now, by (4.5) and by multiplying (4.6) with 4(1-β) we obtain

$$\begin{aligned} F_{\textrm{bond},\beta }(G_{\textrm{o}}) \ge F_{\textrm{bond},\beta }(G_{\textrm{o}})^{-1} 16(1-\beta ) N + (4- 2\beta )\,. \end{aligned}$$

By solving the quadratic equation $t^2-(4-2\beta )t -16(1-\beta ) N \ge 0$ for t≥0, we obtain

4.10 $$\begin{aligned} F_{\textrm{bond},\beta }(G_{\textrm{o}}) \ge \sqrt{(2-\beta )^2 +16(1-\beta ) N} + 2-\beta \,. \end{aligned}$$

Let $\overline{h} = \lceil ((1-\beta ) N)^{1/2}\rceil$, $h_c = ((1-\beta ) N)^{1/2}$, and $f_N(h) = 2\,h + 2(1-\beta ) \frac{N}{h}$. We then have $|h_c-\overline{h}|\le 1$ and using that $\min _h f_N(h) = f_N(h_c)$, by Taylor expansion, together with the fact that $\overline{h} \ge h_c$ and thus $f''_N(h_c)\ge f''_N(h)$ for all $h \in [h_c,\overline{h}]$, we get

$$\begin{aligned} 2\overline{h}+ 2(1-\beta ) \frac{N}{\overline{h}}&= f_N(\overline{h}) \le f_N(h_c) + \frac{1}{2}f_N''(h_c)(\overline{h}-h_c)^2 \\&=4\sqrt{(1-\beta ) N} + 2 (1-\beta )^{-\frac{1}{2}}N^{-\frac{1}{2}}\,. \end{aligned}$$

Therefore, using (4.10), we obtain

$$\begin{aligned} m_\beta (N)&\le 2\overline{h} + 2(1-\beta ) \bigg \lceil \frac{N}{\overline{h}} \bigg \rceil \le 2\overline{h} + 2(1-\beta )\left( \frac{N}{\overline{h}} +1\right) \\  &\le 2(1-\beta ) +4\sqrt{(1-\beta ) N} + 2 (1-\beta )^{-\frac{1}{2}}N^{-\frac{1}{2}} \\  &< - \beta + 2 (1-\beta )^{-\frac{1}{2}}N^{-\frac{1}{2}} +F_{\textrm{bond},\beta }(G_{\textrm{o}}) \,. \end{aligned}$$

For $N \ge N_\beta :=\beta ^{-2} 4 (1-\beta )^{-1}$ this shows (4.4) and concludes the proof.□

### Fluctuation estimates

The goal of this section is to prove Theorem 2.4. Notice that we assume $\beta \in (0,1)$ throughout this section.

#### Definition 4.2

Given $N \in \mathbb {N}$ we define $\mathcal {H}_{\beta ,N} :\{1,\ldots ,N\} \rightarrow \mathbb {R}$ and $\mathcal {L}_{\beta ,N} :\{1,\ldots ,N\} \rightarrow \mathbb {R}$ by

4.11 $$\begin{aligned} \mathcal {H}_{\beta ,N}(h) = 2h + 2(1-\beta ) \bigg \lceil \frac{N}{h} \bigg \rceil \,, \quad \mathcal {L}_{\beta ,N}(l) = 2\bigg \lceil \frac{N}{l} \bigg \rceil + 2(1-\beta ) l\,, \end{aligned}$$

and $$\mathcal {E}_\beta :\mathbb {N} \times \mathbb {N} \rightarrow \mathbb {R}$$ by

$$\begin{aligned} \mathcal {E}_\beta (h,l) = 2h + 2(1-\beta ) l\,. \end{aligned}$$

Moreover, we set

4.12 $$\begin{aligned} h_* =(1-\beta )^{1/2} N^{1/2} \,, \quad l_* = (1-\beta )^{-1/2} N^{1/2} \,. \end{aligned}$$

We observe that

$$\begin{aligned} m_\beta (N)&= \min _h \mathcal {H}_{\beta ,N}(h) =\min _l \mathcal {L}_{\beta ,N}(l) = \min \left\{ \mathcal {E}_\beta (h,l) :h,l \in \mathbb {N}, h\cdot l \ge N \right\} \,, \end{aligned}$$

and

$$\begin{aligned} 2h_* + 2(1-\beta ) \frac{N}{h_*}&= \min _{h>0} \Big (2h + 2(1-\beta ) \frac{N}{h}\Big )\,, \ \ \quad 2\frac{N}{l_*} + 2(1-\beta ) l_* \\&= \min _{l>0} \Big (2\frac{N}{l} + 2(1-\beta ) \Big )\,. \end{aligned}$$

Thus, as the next lemma shows, $h_*$ and $l_*$ provide good reference values for minimizers of $\mathcal {H}_{\beta ,N}$ and $\mathcal {L}_{\beta ,N}$, respectively.

#### Lemma 4.3

There exists a constant $C_\beta >0$ such that for all minimizers $h \in \mathbb {N}$ and $l \in \mathbb {N}$ of $\mathcal {H}_{\beta ,N}$ and $\mathcal {L}_{\beta ,N}$, respectively, we have

4.13 $$\begin{aligned} |h-h_*| \le C_\beta N^{1/4}\,, \quad \quad |l-l_*|\le C_\beta N^{1/4} \,, \end{aligned}$$

where $h_*$ and $l_*$ are given by (4.12). Furthermore, if $\beta \in \mathbb {R} \setminus \mathbb {Q}$, then the minimizers of $\mathcal {H}_{\beta ,N}$ and $\mathcal {L}_{\beta ,N}$ are unique.

#### Proof

Note that the proofs for minimizers of $\mathcal {H}_{\beta ,N}$ and $\mathcal {L}_{\beta ,N}$ are analogous. We therefore only prove the statements for minimizers of $\mathcal {H}_{\beta ,N}$. We first show that for $\beta \in \mathbb {R} \setminus \mathbb {Q}$ the minimizer of $\mathcal {H}_{\beta ,N}$ is unique. To this end, assume by contradiction that there exist two minimizers $h_1,h_2 \in \{1,\ldots ,N\}$. Then,

$$\begin{aligned} 2h_1 + 2(1-\beta )\bigg \lceil \frac{N}{h_1} \bigg \rceil = m_\beta (N) = 2h_2 + 2(1-\beta ) \bigg \lceil \frac{N}{h_2} \bigg \rceil \,, \end{aligned}$$

which is equivalent to $2(h_1-h_2) = 2(1-\beta ) (\lceil \frac{N}{h_2} \rceil - \lceil \frac{N}{h_1} \rceil )$. However, this last equation cannot hold as $2(1-\beta ) \in \mathbb {R}{\setminus } \mathbb {Q}$.

Next, we prove (4.13). Let $h \in \mathbb {N}$ be a minimizer of $\mathcal {H}_{\beta ,N}$ and define $h^* = \lceil h_*\rceil$. Then

$$\begin{aligned} 2h + 2(1-\beta ) \Big \lceil \frac{N}{h} \Big \rceil&=\mathcal {H}_{\beta ,N}(h) \le \mathcal {H}_{\beta ,N}(h^*) = 2 \lceil h_*\rceil + 2(1-\beta ) \bigg \lceil \frac{N}{\lceil h_*\rceil } \bigg \rceil \\  &\le 2(h_*+1) + 2(1-\beta ) \left( \frac{N}{ h_*}+1\right) \\  &= 4-2\beta +\min \left\{ 2h + 2(1-\beta )\frac{N}{h} :h>0 \right\} \\&=4-2\beta + 4\left( (1-\beta ) N\right) ^{\frac{1}{2}}\,. \end{aligned}$$

Multiplying by *h*, we obtain

$$\begin{aligned} 2h^2 -\left( 4-2\beta + 4\left( (1-\beta ) N\right) ^{\frac{1}{2}}\right) h + 2(1-\beta ) N \le 0\,. \end{aligned}$$

The left-hand side is quadratic in *h* with zeros at $h_\pm = \frac{2-\beta }{2} +h_* \pm \frac{1}{4}\left( (4-2\beta )^2 +16(2\right. \left. -\beta )h_*\right) ^\frac{1}{2}$ and thus $h_- \le h \le h_+$. Noting that $h_* \le N^{\frac{1}{2}}$ by (4.12), this implies

$$\begin{aligned} |h-h_*| \le \left( \frac{2-\beta }{2} +\frac{1}{4}32^\frac{1}{2}(2-\beta )^\frac{1}{2}\right) N^\frac{1}{4}\,. \end{aligned}$$

By choosing $C_\beta := \frac{2-\beta }{2} +\frac{1}{4}32^\frac{1}{2}(2-\beta )^\frac{1}{2}$ we conclude the proof.□

We now first prove Theorem 2.4 for $\beta \in \mathbb {Q}$.

*Proof of Theorem *2.4*, upper bound *($U_1$). Let $\beta \in \mathbb {Q}$, $N\ge N_\beta$, with $N_\beta$ from Theorem 2.1, and let $C_N$ be a minimizer of $\mathcal {F}_\beta$. By Theorem 2.1 we have that $C_N \subset \mathbb {Z}^2$. By Lemma 4.3 it suffices to prove that there exists a horizontal translation in $\mathbb {Z} \times \{0\}$ and $C_\beta >0$ such that

4.14 $$\begin{aligned} \#\left( C_{N} \triangle \left( R_{\max }(\beta ,N) +\tau \right) \right) \le C_\beta N^{3/4} \,, \end{aligned}$$

where $$R_{\max }(\beta ,N) =\{1,\ldots ,l_{\max }\}\times \{1,\ldots ,h_{\max }\}$$ with

$$\begin{aligned} l_{\max }:= \max \{l :\mathcal {L}_{\beta ,N}(l) =m_\beta (N)\}\,, \quad h_{\max }:= \max \{h :\mathcal {H}_{\beta ,N}(h) =m_\beta (N)\}\,. \end{aligned}$$

Without loss of generality we can assume that the term on the left-hand side of (4.14) is minimized for τ=0. By Lemma 4.3, Remark 2.2, and Definition 4.2, we have that $C_N \subset R_{\max }(\beta ,N)$. As $\# C_N=N$, it suffices to show that

$$\begin{aligned} \#R_{\max }(\beta ,N) \le N+C_\beta N^{3/4}\,. \end{aligned}$$

Now, by Lemma 4.3 we have that $| h_\textrm{max} -h_*| \le C_\beta N^{1/4}$ and $| l_\textrm{max} -l_*|\le C_\beta N^{1/4}$ with $l_*,h_*$ given in (4.12). As $l_*,h_* \le C_\beta N^{1/2}$ and $l_*\cdot h_*=N$, this shows ($U_1$). □

*Proof of Theorem* 2.4*, lower bound *($L_1$). Let again $\beta \in \mathbb {Q}$. Let $\bar{p},\bar{q} \in \mathbb {N}$, $\bar{p} <\bar{q}$, be such that $1-\beta =\frac{\bar{p}}{\bar{q}}$. Let $0<\delta < \min \{\tfrac{1}{2\bar{p}}, \tfrac{1}{2\bar{q}}\}$ with $\delta ^{-1} \in \mathbb {N}$. Set $p = \delta ^{-2} \bar{p}$ and $q = \delta ^{-2} \bar{q}$. Let $N_k = k^4 pq +(1-\delta )k^2p \in \mathbb {N}$ for each $k \in \mathbb {N}$. We claim that

4.15 $$\begin{aligned} m_\beta (N_k) = 2k^2p + 2(1-\beta )(k^2q+1)\,. \end{aligned}$$

We postpone the proof of (4.15) and show first how we can conclude once (4.15) is proven. Using (4.15) and $1-\beta =\frac{p}{q}$, we have that

$$\begin{aligned} C_{N_k}^1:= \left( \{1,\ldots ,k^2q\}\times \{1,\ldots ,k^2p\}\right) \cup \left( \{k^2q+1\} \times \{1,\ldots ,(1-\delta )k^2p\}\right) \end{aligned}$$

and

$$\begin{aligned} C_{N_k}^2:=&\left( \{1,\ldots ,k^2q(1+\delta ^2\frac{1}{k})\}\times \{1,\ldots ,k^2p(1-\delta ^2\frac{1}{k})\}\right) \\  &\cup \left( \{k^2q(1+\delta ^2\frac{1}{k})+1\}\times \{1,\ldots ,(1-\delta )k^2p +\delta ^4k^2pq\}\right) \end{aligned}$$

are minimizers for $N_k$. (In particular, it is elementary to check that indeed both configurations consist of $N_k$ particles. Here, we also used that $k\delta \bar{q} <k - \delta$.) Furthermore, since $k^3\ge c_\beta N_k^{3/4}$ for some $c_\beta >0$ depending on β, for all $\tau \in \mathbb {Z}\times \{0\}$ we have

$$\begin{aligned} \#(C_{N_k}^1\triangle (C_{N_k}^2+\tau )) \ge c_\beta N_k^{3/4}\,. \end{aligned}$$

This shows that ($L_1$) must be satisfied either for $C_{N_k}^1$ or $C_{N_k}^2$.

It remains to prove (4.15). Testing (2.2) with $h=pk^2$, we obtain $m_\beta (N_k) \le 2k^2p + 2(1-\beta ) (k^2q+1)$. We now need to prove $m_\beta (N_k) \ge 2k^2p + 2(1-\beta )(k^2q+1)$. To this end, observe first that for $N_k^0 :=pqk^4$ we get

4.16 $$\begin{aligned} m_\beta (N_k^0) =2pk^2 + 2(1-\beta ) qk^2\,. \end{aligned}$$

Indeed, we have $h_*=pk^2,l_*=qk^2 \in \mathbb {N}$ (defined in (4.12) for $N_k^0$ in place of *N*) and therefore

$$\begin{aligned} 2pk^2 + 2(1-\beta ) qk^2 = \mathcal {H}_{\beta , N_k^0 }(h_*)&\ge m_\beta (N_k^0) \ge \min _{h>0} \Big ( 2h+ 2(1-\beta ) \frac{ N_k^0 }{h}\Big ) \\  &= 2h_* + 2(1-\beta ) \frac{ N_k^0 }{h_*} = 2pk^2 + 2(1-\beta ) qk^2\,. \end{aligned}$$

This shows (4.16). By the convexity of $h\mapsto \frac{N_k^0}{h}$ we further have

4.17 $$\begin{aligned} \frac{N_k^0}{h} \ge \frac{N_k^0}{h_*} - \frac{N_k^0}{h_*^2} (h-h_*) = \frac{N_k^0}{h_*} - \frac{q}{p} (h-h_*) = \frac{N_k^0}{h_*} - \frac{1}{1-\beta } (h-h_*) \, . \end{aligned}$$

For any minimizer $h \in \{1,\ldots , N_k\}$ it holds that $|h- h_* | \le C_\beta N_k^{1/4}$ by Lemma 4.3. Along with (4.16)–(4.17), for *k* large enough this shows

$$\begin{aligned} 2h + 2(1-\beta ) \bigg \lceil \frac{N_k}{h}\bigg \rceil&= 2 h_* +2(h- h_* ) + 2(1-\beta ) \bigg \lceil \frac{N_k^0}{h}+(1-\delta )\frac{h_*}{h}\bigg \rceil \\  &\ge m_\beta (N_k^0) +2(h-h_*) + 2(1-\beta ) \bigg \lceil \frac{1}{1-\beta } (h_*-h)+(1-\delta )\frac{h_*}{h}\bigg \rceil \\  &\ge m_\beta (N_k^0) +2(h-h_*) + 2(1-\beta ) \bigg \lceil \frac{1}{1-\beta } (h_*-h)+(1-2\delta )\bigg \rceil \,. \end{aligned}$$

Here, we used that $\frac{h_*}{h} \ge 1 - \delta$ for *k* large enough such that $C_\beta N_k^{1/4}\le \frac{1}{2} h_* = \frac{1}{2}(1-\beta )^{1/2}N_k^{1/2}$ and $2 C_\beta (1-\beta )^{-1/2} N_k^{-1/4}\le \delta$, recalling (4.12) and  Lemma 4.3. In view of (4.16), in order to conclude the proof of (4.15), it suffices to show $2(h-h_*) + 2(1-\beta ) \bigg \lceil \frac{1}{1-\beta } (h_*-h)-2\delta \bigg \rceil \ge 0$. Recalling that $1-\beta =\frac{\bar{p}}{\bar{q}}$ and setting $\bar{q}(h-h_*) =\bar{p}l+r$ with $l \in \mathbb {Z}$, $r \in \{0,\ldots ,\bar{p}-1\}$, we have

$$\begin{aligned}&\frac{1}{1-\beta } (h-h_*) + \bigg \lceil \frac{1}{1-\beta } (h_*-h)-2\delta \bigg \rceil = \frac{1}{\bar{p}}(\bar{p}l+r) + \bigg \lceil -\frac{1}{\bar{p}}(\bar{p}l+r) -2\delta \bigg \rceil \\&= \frac{r}{\bar{p}} +\bigg \lceil -\frac{r}{\bar{p}}-2\delta \bigg \rceil \ge 0\,, \end{aligned}$$

because $-\frac{r}{\bar{p}}-2\delta > -\frac{\bar{p}-1}{\bar{p}} -2 \frac{1}{2\bar{p}}= - 1$ as $\delta < \frac{1}{2\bar{p}}$. This shows (4.15) and concludes the proof of ($L_1$). □

We now consider the case that $\beta \in \mathbb {R}\setminus \mathbb {Q}$ is algebraic. Denoting by *N* the number of particles of a configuration and by *h* its height, and by *l* its length, it is not restrictive to reduce the problem to considering configurations where (h-1)-rows consist of *l* particles and the upmost row consists of at most *l* particles, cf. Remark 2.2. Such configurations will be called (*h*, *l*)*-configurations* in the following. If also the upmost row consists of *l* particles, we call such a configuration an (*h*, *l*)*-rectangle*. In the latter case, we have N=hl. Given an (*h*, *l*)-configuration, the corresponding energy is given by $\mathcal {H}_{\beta ,N}(h) = 2\,h + 2(1-\beta ) \lceil \frac{N}{h} \rceil$, as defined in (4.11). We recall by Lemma 4.3 that, due to the fact that β is irrational, the minimizer *h* of $\mathcal {H}_{\beta ,N}$ is unique. Moreover, the energy of different (*h*, *l*)-rectangles is necessarily different.

We fix $N_0 \in \mathbb {N}$ sufficiently large. Our strategy consists in characterizing the optimal (*h*, *l*)-rectangles with particle number *N* with $N \in [N_0,2N_0]$. By Lemma 4.3 we know that the unique optimal height corresponding to *N*, denoted by $h_*(\beta ,N)$ (see (2.3)), is given by

4.18 $$\begin{aligned} h_*(\beta ,N) = \sqrt{1-\beta } N^{1/2} + O(N^{1/4}). \end{aligned}$$

This shows that for particle numbers $$N \in [N_0,2N_0]$$ it suffices to consider heights between $$c_\beta \sqrt{N_0}$$ and $$C_\beta \sqrt{N_0}$$ for some $$0<c_\beta <C_\beta $$, provided that $$N_0$$ is chosen sufficiently large. (For technical reasons we will also consider a slightly larger interval.) Given such a height $$h_0 $$, i.e., $$c_\beta \sqrt{N_0} \le h_0 \le C_\beta \sqrt{N_0}$$, we choose the unique $$l(h_0)$$ such that

$$\begin{aligned} l(h_0) = \textrm{argmin}_l \, \Big | \frac{1}{1-\beta } - \frac{l}{h_0}\Big |. \end{aligned}$$

It is elementary to check that

4.19 $$\begin{aligned} \Big | \frac{1}{1-\beta } - \frac{l(h_0)}{h_0}\Big | \le \frac{1}{h_0}. \end{aligned}$$

In the sequel, we will consider $(h_0,l)$-rectangles, and we want to assess whether they are optimal or not. Optimality corresponds to

$$\begin{aligned} h_0 = \textrm{argmin}_h \, \mathcal {H}_{\beta ,h_0l} (h). \end{aligned}$$

The main properties are the following.

#### Lemma 4.4

For $0<\delta <\frac{1}{6}$ there exists $N_{\beta ,\delta } \in \mathbb {N}$ such that for all $N_0 \ge N_{\beta ,\delta }$ and each $\frac{1}{2} c_\beta \sqrt{N_0} \le h_0 \le 2 C_\beta \sqrt{N_0}$ the following holds:

(i) For all $l \in \mathbb {N}$ with $|l-l(h_0)| \le N_0^{1/6 - \delta }$, the $(h_0,l)$-rectangle is optimal.
(ii) For all $l \in \mathbb {N}$ with $|l-l(h_0)| \ge N_0^{1/6 + \delta }$, the $(h_0,l)$-rectangle is not optimal.

This shows that among configurations with particle number between $N_0$ and $2N_0$ there are at most $\sim N_0^{2/3 + \delta }$ optimal rectangles and at least $\sim N_0^{2/3 - \delta }$ optimal rectangles. To derive a fluctuation estimate from this, we need another property, namely that for each optimal rectangle there is another optimal rectangle whose particle number differs in a quantified way.

#### Lemma 4.5

For $0<\delta <\frac{1}{6}$ there exists $N_{\beta ,\delta } \in \mathbb {N}$ such that for all $N_0 \ge N_{\beta ,\delta }$ the following holds. Let $h,l \in \mathbb {N}$ be such that $hl=N_0$, $c_\beta \sqrt{N_0} \le h \le C_\beta \sqrt{N_0}$, and assume that the (*h*, *l*)-rectangle is optimal. Then,

(i) there exist $h_+,l_+\in \mathbb {N}$ such that the $(h_+,l_+)$-rectangle is optimal and for $N_+ = h_+ l_+$ we have
  $$\begin{aligned} 0< N_+-N_0\le N_0^{1/3+\delta }\,; \end{aligned}$$
(ii) there exist $h_-,l_-\in \mathbb {N}$ such that the $(h_-,l_-)$-rectangle is optimal and for $N_- = h_- l_-$ we have
  $$\begin{aligned} 0< N_0-N_-\le N_0^{1/3+\delta }\,. \end{aligned}$$

We postpone the proofs of Lemma 4.4 and Lemma 4.5 and first explain the fluctuation estimate.

*Proof of the Theorem *2.4*, lower bound *($L_2$)* and upper bound *($U_2$). We start by a preliminary observation. Consider an optimal $(h_0,l)$-configuration consisting of *N* particles with $N < h_0l$. Then, also the $(h_0,l)$-configuration consisting of N+1 particles (adding one particle in the upmost row) is optimal. Indeed, by the monotonicity of the energy in the particle number and the fact that the two configurations have the same energy we get $\min _h \mathcal {H}_{\beta ,N+1}(h) \ge \min _h \mathcal {H}_{\beta ,N}(h) = \mathcal {H}_{\beta ,N}(h_0) = \mathcal {H}_{\beta ,N+1}(h_0)$. This shows that the $(h_0,l)$-configuration with N+1 particles is optimal. Repeating this argument we also find that the $(h_0,l)$-rectangle consisting of $h_0l$ particles is optimal.

In particular, this argument shows that $\min \mathcal {H}_{\beta ,N} < \min \mathcal {H}_{\beta ,N+1}$ is only possible if *N* is a particle number for which an optimal rectangle exists. We now proceed with the proof of the lower bound ($L_2$) and of the upper bound ($U_2$). To this end, we let $N_0 \ge N_{\beta ,\delta }$, where $N_{\beta ,\delta }$ denotes the (maximum of the) constants in Lemma 4.4–Lemma 4.5.

*Proof of the lower bound *($$L_2$$): Since by Lemma 4.4(ii) and the comment below (4.18) there are at most $$ C_\beta N_0^{2/3 + \delta }$$ many optimal rectangles with particle numbers between $$N_0$$ and $$2N_0$$, we can choose $$N \in [N_0,2N_0]$$ such that $$N' - N \ge c_\beta N_0^{1/3-\delta }$$, where $N^{\prime }>N$ denotes the smallest number bigger than *N* such that there exists an optimal rectangle with $N^{\prime }$ particles, and $$ c_\beta >0$$ is a constant depending only on β. The above preliminary observation yields $$\min \mathcal {H}_{\beta ,N'} = \min \mathcal {H}_{\beta ,N}$$. Consider an optimal (*h*, *l*)-rectangle consisting of $N^{\prime }$ particles, and consider the corresponding (*h*, *l*)-configuration consisting of *N* particles by removing $N^{\prime }-N$ particles from the upmost row. Due to $$\min \mathcal {H}_{\beta ,N'} = \min \mathcal {H}_{\beta ,N}$$, this configuration is also optimal. As $$N' - N \ge c_\beta N_0^{1/3-\delta }$$, this configuration allows for a fluctuation of order $$\sim N_0^{1/3-\delta }$$. Since this holds for all $$N_0 \ge N_{\beta ,\delta } $$, the lower bound ($$L_2$$) follows for a constant $$c_{\beta ,\delta }>0$$ depending on β and δ.

*Proof of the upper bound *($$U_2$$): Fix $$N \in [N_0,2N_0]$$ and consider an optimal (*h*, *l*)-configuration consisting of *N* particles. By the above preliminary observation and by Lemma 4.5 we find that the (*h*, *l*)-rectangle with particle number $N^{\prime }=hl$ is optimal and it holds that $$N' - N \le N_0^{1/3+\delta } $$. (Indeed, if $$N' - N > N_0^{1/3+\delta }$$,  Lemma 4.5(ii) (applied for $N^{\prime }$) would imply that there exists $N<N^{\prime \prime }<N^{\prime }$ such that there exists an optimal rectangle with $N^{\prime \prime }$ particles. As $$\min \mathcal {H}_{\beta ,N'} = \min \mathcal {H}_{\beta ,N}$$, we would get $$ \min \mathcal {H}_{\beta ,N'} = \min \mathcal {H}_{\beta ,N''}$$ by monotonicity, which yields a contradiction as the energy for different optimal rectangles is necessarily different.) This shows that at most $$\sim N_0^{1/3+\delta }$$ particles are missing in the upmost row of the optimal (*h*, *l*)-configuration and thus the fluctuation of the configuration is controlled by $$N_0^{1/3+\delta }$$. As this holds for all $$N_0 \ge N_{\beta ,\delta } $$, the upper bound ($$U_2$$) follows for a constant $$C_{\beta ,\delta }>0$$ depending on β and δ. □

We now come to the proofs of Lemma 4.4 and Lemma 4.5.

#### Proof of Lemma 4.4

Let $0<\delta <\frac{1}{6}$. Along the proof we will progressively increase the lower bound $N_{\beta ,\delta }$, depending on δ and β. Fix a $(h_0,l)$-rectangle, and set $N = h_0l$. Given $h \in \mathbb {N}$, we first rewrite the energy $\mathcal {H}_{\beta ,N}$ defined in (4.11) by easily computing

$$\begin{aligned} \mathcal {H}_{\beta ,N}(h)&= 2 h + 2(1-\beta ) \Big \lceil \frac{h_0 l }{h} \Big \rceil = 2h_0 + 2(1-\beta ) l + 2(h-h_0) + 2(1-\beta ) \Big \lceil \frac{h_0 l }{h} - l \Big \rceil \\&= 2h_0 + 2(1-\beta ) l + 2(h-h_0) + 2(1-\beta ) \Big \lceil \frac{l }{h_0}(h_0-h) + \frac{l}{h h_0} (h_0-h)^2 \Big \rceil \\&= \mathcal {H}_{\beta ,N}(h_0) + 2(h-h_0) + 2(1-\beta ) \big \lceil t(h_0,h,l) \big \rceil \,. \end{aligned}$$

where for shorthand we have set

4.20 $$\begin{aligned} t :=t(h_0,h,l) :=\frac{1}{1-\beta } (h_0-h) + \Big ( \frac{l }{h_0} - \frac{1}{1-\beta } \Big ) (h_0-h) + \frac{l}{h h_0} (h_0-h)^2 . \end{aligned}$$

We observe that

4.21 $$\begin{aligned}&\lceil t \rceil \ge \Big \lceil \frac{1}{1-\beta } (h_0-h)\Big \rceil \text { for all { h}} \quad \Rightarrow \quad h_0 = \textrm{argmin}\, \mathcal {H}_{\beta ,N} (h) \text  , \end{aligned}$$

4.22 $$\begin{aligned}&\lceil t \rceil \le \Big \lceil \frac{1}{1-\beta } (h_0-h)\Big \rceil -1 \text { for some { h}} \quad \Rightarrow \quad \text   h_0 \ne \textrm{argmin}\, \mathcal {H}_{\beta ,N} (h) \text {.} \end{aligned}$$

*Proof of *(i)*:* Consider $l \in \mathbb {N}$ with $|l-l(h_0)| \le N_0^{1/6 - \delta }$. In view of (4.21), for fixed *h*, it suffices to show that $\lceil t \rceil \ge \lceil \frac{1}{1-\beta } (h_0-h)\rceil$. Since β is algebraic, by Roth’s theorem, see [55], we find $c=c(\beta ,\delta )>0$ such that

$$\begin{aligned} {\Big | \frac{1}{1-\beta } - \frac{p}{q}\Big | \ge c q^{-2-\delta } } \end{aligned}$$

for all $p \in \mathbb {Z}$ and $q \in \mathbb {N}$. For the choice $q = |h_0-h|$ this yields $| \frac{1}{1-\beta } |h_0-h| - p| \ge c |h-h_0|^{-1-\delta }$ for all $p \in \mathbb {Z}$. Thus, writing $x = h_0 - h$, we get from (4.20)

$$\begin{aligned} t \ge \Big \lfloor \frac{1}{1-\beta } x \Big \rfloor + c |x|^{-1-\delta } + \Big ( \frac{l }{h_0} - \frac{1}{1-\beta } \Big )x + \frac{l}{h h_0} x^2. \end{aligned}$$

By using (4.19) and the fact that $|l-l(h_0)| \le N_0^{1/6-\delta }$ we further get

$$\begin{aligned} t \ge \Big \lfloor \frac{1}{1-\beta } (h-h_0) \Big \rfloor + c |x|^{-1-\delta } - \Big (1 + N_0^{1/6-\delta }) \frac{1}{h_0} |x| + \frac{l}{h h_0} x^2. \end{aligned}$$

It is not restrictive to assume that $h \in [ \frac{1}{2} c_\beta N_0^{1/2}, 2 C_\beta N_0^{1/2}]$ as otherwise *h* cannot be a minimizer, see (4.18). We have $h_0 \in [ \frac{1}{2} c_\beta N_0^{1/2}, 2 C_\beta N_0^{1/2}]$ by assumption and therefore also $l \ge \frac{1}{2} c_\beta N_0^{1/2}$, cf. (4.19). This shows

$$\begin{aligned} c |x|^{-1-\delta }&- \Big (1 + N_0^{1/6-\delta }\Big ) \frac{1}{h_0} |x| + \frac{l}{h h_0} x^2 \ge c |x|^{-1-\delta } - 4 c_\beta ^{-1} N_0^{-1/3-\delta } |x| \\&+ \frac{1}{8} c_\beta C_\beta ^{-2} N_0^{-1/2} x^2. \end{aligned}$$

If $|x| > 32 (C_\beta c_\beta ^{-1})^2 N_0^{1/6-\delta }$, the sum of the last two terms on the right-hand side is positive. If $|x| < ( \frac{c_\beta c}{ 4 } N_0^{1/3+\delta })^{1/(2+\delta )}$, the sum of the first two terms on the right-hand side is positive. Since $32 (C_\beta c_\beta ^{-1})^2 N_0^{1/6-\delta } < ( \frac{c_\beta c}{4} N_0^{1/3+\delta })^{1/(2+\delta )}$ for $N_0 \ge N_{\beta ,\delta }$ for some $N_{\beta ,\delta }$ depending on δ and β, we conclude

$$\begin{aligned} t > \Big \lfloor \frac{1}{1-\beta } (h-h_0) \Big \rfloor . \end{aligned}$$

This shows $\lceil t \rceil \ge \lceil \frac{1}{1-\beta } (h-h_0) \rceil$ and concludes the proof of (i).

*Proof of *(ii)*:* Consider $l \in \mathbb {N}$ with $|l-l(h_0)| \ge N_0^{1/6 + \delta }$. Without restriction we treat the case $l < l(h_0)$ as the other one is completely analogous. We indicate the adaptations at the end of the proof. In view of (4.22), it suffices to find *h* such that the corresponding *t* defined in (4.20) satisfies $\lceil t \rceil \le \lceil \frac{1}{1-\beta } (h_0-h)\rceil - 1$. Choose α with $\frac{1/3 - \delta }{2-4\delta }< \alpha < \frac{1/6 + \delta }{1+3\delta }$. By Lemma 4.6(i) below, we can find $x \in \mathbb {N}$ with $N_0^{\alpha (1-3\delta )} \le x \le N_0^\alpha$ such that $\frac{1}{1-\beta } x - \lfloor \frac{1}{1-\beta } x \rfloor \le N_0^{-\alpha (1-\delta )}$. We set $h = h_0-x$ and aim to show that the corresponding *t* satisfies $\lceil t \rceil \le \lceil \frac{1}{1-\beta } (h_0-h)\rceil - 1$. Note that we can assume without restriction that $l \le C_\beta N_0^{1/2}$ for some $C_\beta >0$ as otherwise the configuration is clearly not optimal. Using also $h,h_0 \ge \frac{1}{2} c_\beta N_0^{1/2}$, cf. below (4.18), we estimate

$$\begin{aligned} { t \le \Big \lfloor \frac{1}{1-\beta } (h_0-h) \Big \rfloor + N_0^{-\alpha (1-\delta )} + \Big ( \frac{l }{h_0} - \frac{1}{1-\beta } \Big ) x + 4 C_\beta c_\beta ^{-2} N_0^{-1/2} x^2. } \end{aligned}$$

Using x>0, $\frac{l }{h_0} - \frac{1}{1-\beta } \le (- N_0^{1/6+\delta }+1) h_0^{-1}$ (see (4.19) and use the choice of *l*), as well as $h_0 \le 2 C_\beta N_0^{1/2}$ we get

$$\begin{aligned} t \le \Big \lfloor \frac{1}{1-\beta } (h_0-h) \Big \rfloor + N_0^{-\alpha (1-\delta )} - \frac{1}{ 4 } C_\beta ^{-1} N_0^{-1/3+\delta } x + 4 C_\beta c_\beta ^{-2} N_0^{-1/2} x^2, \end{aligned}$$

provided that $N_{\beta ,\delta }$ is chosen sufficiently large. Recalling that $N_0^{\alpha (1-3\delta )} \le x \le N_0^\alpha$ and $\frac{1/3 - \delta }{2-4\delta }< \alpha < \frac{1/6 + \delta }{1+3\delta }$, for $N_0 \ge N_{\beta ,\delta }$ and $N_{\beta ,\delta }$ sufficiently large, we find by an elementary computation that

$$\begin{aligned} t \le \Big \lfloor \frac{1}{1-\beta } (h_0-h) \Big \rfloor . \end{aligned}$$

This yields $\lceil t \rceil \le \lceil \frac{1}{1-\beta } (h_0-h)\rceil - 1$ and concludes the proof.

In the case $l > l(h_0)$, we use Lemma 4.6(ii) below to find $x \in \mathbb {N}$ with $N_0^{\alpha (1-3\delta )} \le x \le N_0^\alpha$ and $\frac{1}{1-\beta } (- x) - \lfloor \frac{1}{1-\beta } (-x) \rfloor \le N_0^{-\alpha (1-\delta )}$, and define $h = h_0 + x$. Then, the statement follows by repeating the estimates above.□

We check the following result which has been used in the previous proof and will be also instrumental in the proof of Lemma 4.5 below.

#### Lemma 4.6

Let $0<\delta <\frac{1}{6}$ and 0<α<1. There exist $N_{\beta ,\delta } \in \mathbb {N}$ (depending on δ, α, and β) and c>0 (depending on δ and β) such that for $N_0 \ge N_{\beta ,\delta }$

(i) we find $x \in \mathbb {N}$ with $N_0^{\alpha (1-3\delta )} \le x \le N_0^\alpha$ such that
  4.23 $$\begin{aligned} c N_0^{-\alpha (1+\delta )} \le \frac{1}{1-\beta } x - \left\lfloor \frac{1}{1-\beta } x \right\rfloor \le N_0^{-\alpha (1-\delta )}\,; \end{aligned}$$
(ii) we find $x \in \mathbb {N}$ with $N_0^{\alpha (1-3\delta )} \le x \le N_0^\alpha$ such that
  $$\begin{aligned} c N_0^{-\alpha (1+\delta )} \le \bigg \lceil \frac{1}{1-\beta } x \bigg \rceil - \frac{1}{1-\beta } x \le N_0^{-\alpha (1-\delta )}\,. \end{aligned}$$

#### Proof

First we show (i). We define the *m*-discrepancy of the sequence $(j \frac{1}{1-\beta } \, \textrm{mod} \, 1)_{j \in \mathbb {N}}$ with respect to the interval [0, *s*], s>0, by

$$\begin{aligned} {\phi _m(s):= \frac{1}{m} \sum _{j=0}^{m-1} \chi _{\mathbb {N}+ [0,s]} (j \tfrac{1}{1-\beta }) -s\,.} \end{aligned}$$

As β is algebraic, by [43, Thm. 3.2 and Ex. 3.1, pp. 123-124], for δ>0 there exists $C_{\delta ,\beta }>0$ depending on δ and β such that

$$\begin{aligned} \sup _s |\phi _m(s)| \le C_{\delta ,\beta } m^{-1+\delta /2} \quad \text {for all } m \in \mathbb {N}. \end{aligned}$$

With $s = N_0^{-\alpha (1-\delta )}$ and $m = \lfloor N_0^{\alpha } \rfloor$ we get

$$\begin{aligned} {\frac{1}{m} \sum _{j=0}^{m-1} \chi _{\mathbb {N}+ [0,s]} (j \tfrac{1}{1-\beta }) \ge N_0^{-\alpha (1-\delta )} - C_{\delta ,\beta } m^{-1+\delta /2} >0\,, } \end{aligned}$$

provided that $N_0 \ge N_{\beta ,\delta }$ for some $N_{\beta ,\delta }$ depending on δ, α, and β. This shows that there exists $x \in \mathbb {N}_0$ such that $x \le N_0^\alpha$ and $\frac{1}{1-\beta } x - \lfloor \frac{1}{1-\beta } x \rfloor \le s= N_0^{-\alpha (1-\delta )}$, i.e., the upper bound in (4.23) holds. By Roth’s Theorem [55] there exists c>0 depending on δ and β such that $| q \frac{1}{1-\beta } - p | > \frac{c}{q^{1+\delta }}$ for all $p \in \mathbb {Z}$ and $q \in \mathbb {N}$. Thus, $| \frac{1}{1-\beta } x - \lfloor \frac{1}{1-\beta } x \rfloor | > \frac{c}{x^{1+\delta }} \ge cN_0^{-\alpha (1+\delta )}$ which yields the lower bound in (4.23). Furthermore, as this also implies $\frac{x^{1+\delta }}{c} \ge N_0^{\alpha (1-\delta )}$ by the upper bound in (4.23), we obtain $x \ge N_0^{\alpha (1-3\delta )}$ for $N_0 \ge N_{\beta ,\delta }$, provided that $N_{\beta ,\delta }$ is chosen sufficiently large.

The proof of (ii) follows along the same lines by using the *m*-discrepancy of the sequence $(j\frac{1}{1-\beta } \, \textrm{mod} \, 1)_{j \in \mathbb {N}}$ with respect to the interval [1-s,1], s>0, namely

$$\begin{aligned} \hat{\phi }_m(s):= \frac{1}{m} \sum _{j=0}^{m-1} \chi _{\mathbb {N}+ [1-s,1]} (j \tfrac{1}{1-\beta }) -s\,. \end{aligned}$$

Using again [43] we can control $\hat{\phi }_m$ in the same way as $\phi _m$ above. □

We prove now Lemma 4.5.

#### Proof of Lemma 4.5

Let $h,l \in \mathbb {N}$, $N_0 =hl$, and suppose that the (*h*, *l*)-rectangle is optimal. We only prove (i) as the proof of (ii) is completely analogous and we briefly indicate the necessary adaptations at the end of the proof. Let $\sigma _l = 1$ if l-l(h)≥0 and $\sigma _l = -1$ else. We apply Lemma 4.6 ((i) or (ii), depending on the sign of $\sigma _l$) for $\alpha = \frac{1}{6}-3\delta$ to choose $p,q \in \mathbb {N}$ with

4.24 $$\begin{aligned} N_0^{(\frac{1}{6}-3\delta )(1-3\delta )} \le q \le N_0^{\frac{1}{6}-3\delta } \quad \text {and}\quad c q\le p \le C q \end{aligned}$$

such that

4.25 $$\begin{aligned} c N_0^{-(\frac{1}{6}-3\delta )(1+\delta )} \le \sigma _l \Big ( q \frac{1}{1-\beta } - p \Big ) \le N_0^{-(\frac{1}{6}-3\delta )(1-\delta )}\,. \end{aligned}$$

We claim that

4.26 $$\begin{aligned} cN_0^{-\frac{1}{3}+ 5 \delta }\le \sigma _l \Big ( \frac{l}{h} - \frac{p}{q} \Big ) \le CN_0^{-\frac{1}{3} + 7 \delta }\,. \end{aligned}$$

Indeed, by (4.19), (4.24), (4.25), and the fact that $|l - l(h)| \le N_0^{1/6+\delta }$ (see Lemma 4.4(ii)), we obtain

$$\begin{aligned} \sigma _l \Big ( \frac{l}{h} - \frac{p}{q} \Big )&\le \frac{1+N_0^{1/6+\delta }}{h} + \sigma _l \Big ( \frac{1}{1-\beta } - \frac{p}{q} \Big ) \le \frac{CN_0^{1/6+\delta }}{h} + N_0^{-(\frac{1}{6}-3\delta )(1-\delta )} q^{-1} \\&\le C N_0^{-1/3+\delta } + C N_0^{-1/3 + \frac{20}{3}\delta -12\delta ^2 } \le CN_0^{-1/3 + 7 \delta }\,, \end{aligned}$$

where the constant depends on δ and β. Here, we used that $h \ge c_\beta N_0^{1/2}$. On the other hand, using again $h \ge c_\beta N_0^{1/2}$,

$$\begin{aligned} \sigma _l \Big ( \frac{l}{h} - \frac{p}{q} \Big )&\ge -\frac{1+N_0^{1/6+\delta }}{h} + \sigma _l \Big ( \frac{1}{1-\beta } - \frac{p}{q} \Big ) \ge -\frac{CN_0^{1/6+\delta }}{h} + cN_0^{-(\frac{1}{6}- 3 \delta )(1+\delta )} q^{-1} \\&\ge -CN_0^{-1/3+\delta } + C N_0^{-1/3 + \frac{35}{6}\delta +3\delta ^2 } \ge CN_0^{-1/3 + 5 \delta }\,. \end{aligned}$$

This shows (4.26). Recalling that $|l-l(h)| \le N_0^{1/6+\delta }$ and $p \le Cq \le C N_0^{1/6-3\delta }$, we can further choose $k \in \mathbb {N}$, $k \le C N_0^{1/6+\delta }/q$, such that

4.27 $$\begin{aligned} \big | 2(1-\beta ) |l-l(h)| - 2kq - 2(1-\beta ) kp \big | \le CN_0^{1/6-3\delta } \end{aligned}$$

for some universal C>0. We now define $l_+ = l - \sigma _l kp$, $h_+ = h + \sigma _l kq$, and $N_+=l_+h_+$. We claim that

4.28 $$\begin{aligned} 0 < N_+-N_0 \le C N_0^{\frac{1}{3} + 8 \delta }\,, \end{aligned}$$

provided that $N_{\beta ,\delta }$ is sufficiently large. Indeed,

$$\begin{aligned} N_+-N_0= h_+l_+ -hl = (h + \sigma _l kq)(l - \sigma _l kp) = \sigma _l \big ( k ql-k h p \big ) -k^2pq . \end{aligned}$$

By (4.26) we find

$$\begin{aligned} kq \big (c h N_0^{-\frac{1}{3}+ 5 \delta } - kp\big )\le \sigma _l \big ( k ql-k h p \big ) -k^2pq \le CN_0^{-\frac{1}{3} + 7 \delta } k q h -k^2 pq\,. \end{aligned}$$

Using that $c_\beta N_0^{\frac{1}{2}}\le h \le C_\beta N_0^{1/2}$, and that $kp, kq \le CN_0^{1/6+\delta }$ we get

$$\begin{aligned} 0<kq (cc_\beta N_0^{\frac{1}{6}+ 5 \delta } -CN_0^{\frac{1}{6}+ \delta } )\le N_+ - N_0 \le CC_\beta N_0^{\frac{1}{3} + 8 \delta }\,, \end{aligned}$$

where the first inequality holds for $N_0\ge N_{\beta ,\delta }$ for some $N_{\beta ,\delta }$ sufficiently large depending on δ and β. This concludes the proof of (4.28). We observe that the desired estimate follows from (4.28) by replacing δ with δ/8.

It remains to check that also the $(h_+, l_+)$-rectangle is optimal. First note that $c_\beta N_0^{1/2} \le h \le C_\beta N_0^{1/2}$ implies $\frac{c_\beta }{2} N_0^{1/2} \le h_+ \le 2C_\beta N_0^{1/2}$ for $N_0 \ge N_{\beta ,\delta }$ and $N_{\beta ,\delta }$ sufficiently large as $kq \le C N_0^{1/6+\delta }$. Thus, in view of  Lemma 4.4(i), it suffices to check that

4.29 $$\begin{aligned} |2 h_+ - 2(1-\beta ) l_+| \le \frac{1}{2}N_0^{1/6- 2 \delta }, \end{aligned}$$

as then, by using (4.19), we get

$$\begin{aligned}&2(1-\beta ) |l_+ - l(h_+)| \le | 2(1-\beta ) l_+ - 2h_+| + | 2(1-\beta ) l(h_+) - 2h_+| \le \frac{1}{2}N_0^{1/6- 2 \delta } \\&\quad +2\le 2(1-\beta ) N_{ \delta }^{1/6-\delta }\,, \end{aligned}$$

where the last inequality holds for $N_{\beta ,\delta }$ sufficiently large depending on δ and β. Let us finally show (4.29). By (4.19) and (4.27), we calculate

$$\begin{aligned} \big |2 h_+ - 2(1-\beta ) l_+\big |&= \big |2 h - 2(1-\beta ) l(h) + 2(1-\beta ) (l(h) - l) + 2 (h_+ - h) - 2(1-\beta ) (l_+-l) \big | \\&\le 2 + \big | 2(1-\beta ) (l(h) - l) + 2kq \sigma _l + 2(1-\beta ) kp \sigma _l \big | \\&\le 2 + CN_0^{1/6- 3 \delta } \le \frac{1}{2}N_0^{1/6 - 2 \delta } \end{aligned}$$

for $N_0\ge N_{\beta ,\delta }$ and $N_{\beta ,\delta }$ sufficiently large depending on δ. Here, we used that $\sigma _l = 1$ if l-l(h)≥0 and $\sigma _l = -1$ else. This shows (4.29) and concludes the proof.

Finally, the proof of (ii) follows along the same lines by choosing *p* and *q* such that (4.25) is replaced by $c N_0^{-(\frac{1}{6}-3\delta )(1+\delta )} \le \sigma _l ( -q \frac{1}{1-\beta } + p ) \le N_0^{-(\frac{1}{6}-3\delta )(1-\delta )}$. □

## Data Availability

Data sharing not applicable to this article as no datasets were generated or analysed during the current study.

## Proof of Lemma 3.2

### Proof of Lemma 3.2

Let $C_n$ be a minimizer of (2.1). For simplicity, we write G=(V,E) instead of $G_{\textrm{nat}}=(V,E_{\textrm{nat}})$ for the associated natural bond graph.

Step 1. We define $V_\textrm{sub} = V \cup \bigcup _{x\in V} \mathcal {N}_{\mathcal {L}^-}(x)$ and $E_\textrm{sub} = E \cup \lbrace \lbrace x,y \rbrace :x \in V, y\in \mathcal {N}_{\mathcal {L}^-}(x) \rbrace$. In this step, we show

A.1 $$\begin{aligned} |x-y| \ge 1-\varepsilon \text { for all } \lbrace x,y \rbrace \in E_\textrm{sub}\,. \end{aligned}$$

We define

A.2 $$\begin{aligned} M:= \max _{x \in \mathbb {R}^2} \#(V_\textrm{sub} \cap B_{\frac{1}{2}(1-\varepsilon )}(x))\,, \end{aligned}$$

where $B_r(x)$ denotes the closed ball with radius r>0 centered at *x* and we write $B_r$ in the following whenever x=0. Claim (A.1) will follow by showing M=1. Let $x_0 \in \mathbb {R}^2$ be a maximizer in (A.2). After translation of $V_\textrm{sub}$, it is not restrictive to assume that $x_0=0$. As $\#\{\{x,y\} \in E_\textrm{sub} :x,y \in B_{\frac{1}{2}(1-\varepsilon )}\} \ge \frac{M(M-1)}{2}$, by assumption ($\textrm{iii}_2$), letting $\lambda = \min \{1, 2 \beta \}$, we have

A.3 $$\begin{aligned} \underset{\{x,y\} \in E}{\sum _{x,y \in B_{\frac{1}{2}(1-\varepsilon )}}} v_2(|x-y|) + 2 \beta \underset{x \in V, y\in \mathcal {N}_{\mathcal {L}^-}(x) }{\sum _{x,y\in B_{\frac{1}{2}(1-\varepsilon )} }} v_2(|x-y|) \ge \frac{\lambda }{2\varepsilon }M(M-1)\,. \end{aligned}$$

Consider the annulus $A_\varepsilon :=B_{\frac{1}{2}(1-\varepsilon ) +\sqrt{2}} \setminus B_{\frac{1}{2}(1-\varepsilon )} \subset B_{\frac{1}{2}+\sqrt{2}}$. By covering $B_{\frac{1}{2}+\sqrt{2}}$ with discs of radius $\frac{1}{4}$ we get that there exists $K \in \mathbb {N}$ and $\{z_i\}_{i=1}^{K} \subset \mathbb {R}^2$ such that for all $0 < \varepsilon \le \frac{1}{2}$

$$\begin{aligned} A_\varepsilon \subset B_{\frac{1}{2}+\sqrt{2}} \subset \bigcup _{i=1}^{K} B_{\frac{1}{4}}(z_i) \subset \bigcup _{i=1}^{K} B_{\frac{1}{2}(1-\varepsilon )}(z_i)\,. \end{aligned}$$

Note that *K* is independent of ε (provided $\varepsilon <\frac{1}{2}$). Recalling (A.2), we have

A.4 $$\begin{aligned} \#(V_\textrm{sub} \cap A_\varepsilon ) \le \#\left( V_\textrm{sub}\cap \bigcup _{i=1}^K B_{\frac{1}{2}(1-\varepsilon )}(z_i)\right) \le \sum _{i=1}^K \#\left( V_\textrm{sub} \cap B_{\frac{1}{2}(1-\varepsilon )}(z_i)\right) \le K M\,. \end{aligned}$$

By (A.4), the definition of *M*, and ($\mathrm i_2$), letting $\Lambda = \max \{1, \beta \}$ we have

A.5 $$\begin{aligned}&-\underset{\{x,y\} \in E}{\sum _{x \in B_{\frac{1}{2}(1-\varepsilon )}, y \in A_\varepsilon }} v_2(|x-y|) - \beta \underset{x \in V, y\in \mathcal {N}_{\mathcal {L}^-}(x) }{\sum _{x \in B_{\frac{1}{2}(1-\varepsilon )}, y \in A_\varepsilon }} v_2(|x-y|)&\le \Lambda \cdot \#\{x \in V_\textrm{sub} \cap B_{\frac{1}{2}(1-\varepsilon )}, \nonumber \\&y \in V_\textrm{sub} \cap A_\varepsilon \} \le \Lambda KM^2\,. \end{aligned}$$

We write $V\cap B_{\frac{1}{2}(1-\varepsilon )} = \{x_i\}_{i=1}^{\bar{M}}$, $\bar{M}\le M$, and consider a competitor $\hat{V}$ (with associated natural bond graph $\hat{G}$) given by

$$\begin{aligned} \hat{V} = (V \setminus B_{\frac{1}{2}(1-\varepsilon )}) \cup \bigcup _{i=1}^M \{x_i +\tau _i\}\,, \end{aligned}$$

where $\tau _i \in \mathbb {R}^2$ are chosen such that

A.6 $$\begin{aligned} \textrm{dist}(x_i+\tau _i, \hat{V}\setminus \{x_i+\tau _i\}) \ge \sqrt{2} \text { for all } i=1,\ldots , \bar{M}\,. \end{aligned}$$

By (3.4), (A.6), ($\mathrm ii_2$), and the optimality of *G* we have

A.7 $$\begin{aligned} F_\beta (G) \le F_\beta (\hat{G})&\le F_\beta (G) - \underset{\{x,y\} \in E}{\sum _{x,y \in B_{\frac{1}{2}(1-\varepsilon )}}} v_2(|x-y|)- 2 \beta \underset{x \in V, y\in \mathcal {N}_{\mathcal {L}^-}(x) }{\sum _{x,y\in B_{\frac{1}{2}(1-\varepsilon )} }} v_2(|x-y|) \nonumber \\&\quad - 2 \underset{\{x,y\} \in E}{\sum _{x \in B_{\frac{1}{2}(1-\varepsilon )}, y \in A_\varepsilon }} v_2(|x-y|) - 2 \beta \underset{x \in V, y\in \mathcal {N}_{\mathcal {L}^-}(x) }{\sum _{x \in B_{\frac{1}{2}(1-\varepsilon )}, y \in A_\varepsilon }} v_2(|x-y|) \,. \end{aligned}$$

Now, using (A.3), (A.5), and (A.7), we obtain

$$\begin{aligned} \frac{\lambda }{2\varepsilon }M(M-1) \le 2 \Lambda K M^2\,. \end{aligned}$$

For ε>0 small enough ($\varepsilon < \frac{\lambda }{5\Lambda K }$ suffices), this inequality can only be true for M=1. This yields (A.1) and concludes Step 1.

Step 2. In this step we prove that all bond angles satisfy

A.8 $$\begin{aligned} \theta \in [\pi /2-\varepsilon ,\pi /2+\varepsilon ] \cup [\pi -\varepsilon ,\pi +\varepsilon ] \cup [3\pi /2-\varepsilon ,3\pi /2+\varepsilon ]\,. \end{aligned}$$

In particular, by choosing $\varepsilon < \frac{1}{10}\pi$, this will also imply that $\#\mathcal {N}(x,E) \le 4$ and $\# \mathcal {N}_{\mathcal {L}^-}(x) \le 1$ for all x∈V, where we recall that in (2.1) the energy contributions is due to triples $(x,y,z) \in \left( C_N\cup \mathcal {L}^-\right) ^3$. To see (A.8), we first claim that

A.9 $$\begin{aligned} \#\mathcal {N}(x,E) + \# \mathcal {N}_{\mathcal {L}^-}(x) \le 4\frac{\left( \sqrt{2} +\frac{1}{2}\right) ^2}{(1-\varepsilon )^2} \text { for all } x\in V\,. \end{aligned}$$

Indeed, by Step 1, ($\textrm{ii}_2$), and by the fact that $B_{\frac{1}{2}(1-\varepsilon )}(y) \subset B_{\sqrt{2}+\frac{1}{2}}(x)$ for all $y \in \mathcal {N}(x,E) \cup \mathcal {N}_{\mathcal {L}^-}(x)$ we have

$$\begin{aligned} \big (\sqrt{2} +\tfrac{1}{2}\big )^2\pi&= \big | B_{\sqrt{2}+\frac{1}{2}}(x)\big | \ge \sum _{y \in \mathcal {N}(x,E) \cup \mathcal {N}_{\mathcal {L}^-}(x) } \big |B_{\frac{1}{2}(1-\varepsilon )}(y)\big | \\&\ge \frac{1}{4}(1-\varepsilon )^2 \pi \, \big (\# \mathcal {N}(x,E) + \# \mathcal {N}_{\mathcal {L}^-}(x) \big ) \,, \end{aligned}$$

i.e., (A.9) holds. We now show (A.8). In fact, assuming by contradiction that *x* has a bond angle $\theta _{y,x,z}$ that does not satisfy (A.8), we could define $\hat{V}= (V{\setminus } \{x\} )\cup \{x+\tau \}$ for some $\tau \in \mathbb {R}^2$ such that $\textrm{dist}(x+\tau , \hat{V}{\setminus } \{x+\tau \})\ge \sqrt{2}$. Then, by ($\mathrm i_2$), ($\mathrm iv_3$), and (A.9) we obtain a contradiction to the minimality of *G*, namely

$$\begin{aligned} F_\beta (\hat{G}) \le F_\beta (G) + \max \{1, 2\beta \} \big ( \#\mathcal {N}(x,E) + \# \mathcal {N}_{\mathcal {L}^-}(x) \big )- v_3(\theta _{y,x,z}) <F_\beta (G)\,. \end{aligned}$$

Summarizing, with choosing $\varepsilon _0: = \min \{\frac{1}{10}\pi , \frac{\lambda }{8K\Lambda } \}$, the statement holds. □
